# Microbial Electrosynthesis Reshapes Energy Metabolism and Physiology in *Clostridium ljungdahlii*

**DOI:** 10.1111/1751-7915.70398

**Published:** 2026-06-06

**Authors:** Sara Al Sbei, Santiago T. Boto, Thomas Krüger, Kai Papenfort, Martin Westermann, Aurélie Jost, Falk Harnisch, Axel A. Brakhage, Miriam A. Rosenbaum

**Affiliations:** ^1^ Bio Pilot Plant Leibniz Institute for Natural Product Research and Infection Biology–Hans‐Knöll‐Institute Jena Germany; ^2^ Institute of Microbiology, Faculty of Biological Sciences Friedrich Schiller University Jena Jena Germany; ^3^ Molecular and Applied Microbiology Leibniz Institute for Natural Product Research and Infection Biology–Hans‐Knöll‐Institute Jena Germany; ^4^ Cluster of Excellence Balance of the Microverse Friedrich‐Schiller‐University Jena Jena Germany; ^5^ Electron Microscopy Center Jena University Hospital Jena Germany; ^6^ Microverse Imaging Center Friedrich‐Schiller‐University Jena Jena Germany; ^7^ Department of Microbial Biotechnology UFZ‐Helmholtz Centre for Environmental Research Leipzig Germany

**Keywords:** bacterial microcompartments (BMCs), *Clostridium ljungdahlii*, cyanophycin, glycine synthase‐reductase pathway (GSRP), membrane depolarization, microbial electrosynthesis (MES), Wood‐Ljungdahl pathway

## Abstract

Microbial electrosynthesis (MES) enables a variety of microorganisms, particularly acetogens, to utilize electrical energy in the form of electrons to produce valuable compounds from CO_2_. In the closely related process of gas fermentation, hydrogen gas (H_2_) is provided as the energy source, whereas in MES, H_2_ is produced in situ via water electrolysis. Despite the potential of MES for energy and carbon storage, it still faces major limitations, like low efficiency and low‐value products. Here, we identify key limitations of the model MES biocatalyst *Clostridium ljungdahlii* through comparative transcriptomics, proteomics, and electron microscopy in both processes. We show that cell integrity is severely impaired in MES, consistent with membrane depolarization hampering ATP synthesis. The struggle for ATP is compensated for by activating arginine catabolism to produce ATP, a reaction that is likely fueled by cyanophycin degradation. Diversion of the Wood‐Ljungdahl pathway toward the glycine synthase‐reductase pathway (GSRP) resulted in a broader spectrum of reduced products, including the two amino compounds ethanolamine and glycine, which appeared exclusively under the electrochemical environment. Additionally, we observed strong induction of bacterial microcompartments, raising questions about their role during MES. This work demonstrates that MES drives *C. ljungdahlii* into a distinct physiological state that challenges cellular fitness and expands our understanding of MES.

## Graphical abstract

Microbial electrosynthesis drives *Clostridium ljungdahlii* into a distinct stress‐associated physiological state, different from gas fermentation. Comparative multi‐omics and electron microscopy reveal physiological constraints that help explain the poor performance of MES.

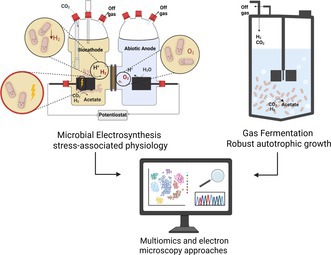

## 1 Introduction

Microbial electrosynthesis (MES) has attracted increasing interest as a microbial platform for converting CO_2_ and electrical energy into organic compounds. In MES, microbes receive electrons from an external electric circuit to reduce CO_2_ to organic compounds (Jourdin and Burdyny 2021; Nevin et al. 2010). MES can be performed by different groups of microorganisms and may involve different electron‐uptake mechanisms, depending on the organism and reactor conditions. These include direct electron transfer as well as indirect transfer via electrochemically generated intermediates such as H_2_ (Rabaey and Rozendal 2010). In the MES system studied here with the acetogen *C. ljungdahlii* , cathodically generated H_2_ is the dominant electron donor supporting CO_2_ fixation via the Wood–Ljungdahl pathway (Boto et al. 2023). This in situ H_2_ feed is a highly promising solution for overcoming gas–liquid mass transfer limitations in processes such as gas fermentation (GF), where H_2_ gas is used as a feedstock (Kracke et al. 2021). MES also offers a potential solution for capturing and storing renewable electrical energy as liquid chemical compounds (Chu et al. 2020). However, despite significant research efforts over the last decade, MES development faces critical bottlenecks, including low microbial biomass and product yields, an economically unattractive product portfolio, and challenges in reactor scale‐up (Boto et al. 2025; Jourdin and Burdyny 2021; Prévoteau et al. 2020).

MES research has predominantly focused on engineering aspects, such as electrode materials, reactor design, and technical operation, whereas the role of the microbial key players, the “bioelectrocatalysts,” has been widely neglected (Boto et al. 2023). One reason is the widespread use of mixed cultures. Using mixed cultures is cost‐effective and robust against toxic oxygen effects; however, it leads to a minimal product portfolio and limits a deeper understanding of the involved microbial physiology, which hinders the advancement of the biological component (Harnisch et al. 2024). The physiology of anodic model organisms like *Shewanella oneidensis* or *Geobacter sulfurreducens* has been intensively studied (Gralnick and Bond 2023). In contrast, model microorganisms for cathodic electron uptake, such as *Clostridium ljungdahlii* , still lack a deeper physiological investigation. Unlike classical model organisms, *C. ljungdahlii* remains difficult to genetically manipulate, which limits rapid functional validation of candidate genes and makes comparative multi‐omics one of the most informative approaches currently available for this system. Moreover, acetogens grow close to the thermodynamic edge of life even under favourable autotrophic conditions, and growth during MES is particularly poor, making physiological investigations under MES especially challenging. As a result, the physiology of *C. ljungdahlii* during MES has largely remained unresolved. Instead, a physiology similar to that during GF is simply assumed, supported by recent research, which disproved the initial hypothesis of a direct electron‐uptake pathway in *C. ljungdahlii* and instead confirmed that H_2_, produced in situ via water electrolysis, is the primary electron donor in MES (Boto et al. 2023). MES is usually performed using the so‐called H‐type reactors. This lab‐scale setup consists of two electrochemical compartments, separated by a cation exchange membrane that separates the cathodic hydrogen evolution from the anodic oxygen evolution reaction (Jourdin et al. 2015). However, the membrane cannot completely shield the obligate anaerobic biocatalyst at the cathode from anodic oxygen (Abdollahi et al. 2022). Our recent work on *C. ljungdahlii* has shown that, in addition to oxygen stress, insufficient H_2_ availability can limit MES performance (Kuchenbuch et al. 2025). We hypothesize that, beyond traditional process engineering factors such as nutrient supply, the electrochemical environment itself may be another critical factor shaping microbial performance during MES. This understudied aspect may alter microbial physiology by affecting cellular redox balances and membrane potentials.

To gain a more thorough understanding of how MES affects the physiological and metabolic state of the model acetogen *C. ljungdahlii* , we compared *C. ljungdahlii* grown under MES and gas fermentation conditions. We used a multi‐omics approach combining transcriptomics and proteomics, together with Transmission Electron Microscopy (TEM) imaging. Our analysis reveals that MES triggers distinct metabolic shifts in *C. ljungdahlii* , with stress responses detectable at multiple levels and identifies a distinct physiological state that highlights the need for MES strategies that better protect the biocatalyst from system‐induced stress.

## 2 Materials and Methods

### 2.1 Bacterial Strain and Cultivation in Microbial Electrosynthesis Reactors and Gas Fermentation

*Clostridium ljungdahlii* DSM 13528 (DSMZ, Germany) was cultivated heterotrophically in reinforced Clostridial medium (RCM) for two sequential overnight transfers at 37°C, followed by transfer to autotrophic conditions in PETC medium. All procedures were performed in a sterile, anaerobic chamber (Coy Laboratory Products, Grass Lake, USA). The pH was measured using a Mettler‐Toledo pH/ion analyser (Gießen, Germany). Optical density at 600 nm was measured with an Eppendorf Biophotometer (Hamburg, Germany).

PETC medium was rendered anaerobic using the same boiling and N_2_‐purging procedure described above, and the medium was distributed into 250 mL serum bottles (30 mL liquid volume). Autotrophic pre‐cultures were grown in PETC medium under a 1.5 bar overpressure CO_2_/H_2_ gas (20:80 Air Liquide, Paris, France) and incubated horizontally at 37°C for 6 days. These pre‐cultures were then used to inoculate MES and GF bioreactor experiments. All chemicals used were of analytical and biochemical purity and supplied by Thermo Fisher Scientific (USA), Merck (Germany), or Carl Roth (Germany). Details about media components can be found in the SI.

For microbial electrosynthesis, we used H‐type reactors with two chambers separated by a cation‐exchange membrane (Laborglas Lammek, Moers, Germany; CMI‐7000S membrane from Membranes International, Ringwood, USA). Graphite EDM‐3 blocks were used as both working and counter electrodes, with a surface area of 26 cm^2^ (Novotec, Reuver, Netherlands). Each chamber of the H‐type reactor was filled with 400 mL of PETC media. To maintain the anaerobic environment in the reactors, they were continuously flushed with a humidified 80:20 N_2_:CO_2_ gas mixture (Air Liquide, Paris, France) at a flow rate of 0.1 L min^−1^. Stirring was performed at 200 rpm, and the reactors were maintained at 37°C to achieve optimal growth of *C. ljungdahlii* . Then, reactors were inoculated with 30 mL of the pre‐cultures. After inoculation, reactors were operated potentiostatically at −0.9 V versus Ag/AgCl (sat KCl) at the cathode, and the corresponding current of electrolytic H_2_ production was recorded (i.e., using chronoamperometry). The cathode potential of −0.9 V versus Ag/AgCl was selected based on previous electrochemical characterization of the system, which showed that this potential provided sufficiently high H_2_ evolution rates to support robust acetogenic growth and product formation under the tested MES conditions (Boto et al. 2023). Samples of 2 mL were collected daily for OD_600_ and HPLC measurements, and biomass for omics analyses was collected during the active growth phase to capture the primary physiological response to MES before prolonged cultivation could obscure early transcriptional changes.

Gas fermentation was carried out in a 10 L ProREACT stirred‐tank bioreactor 3P‐ATEX (Heinrich Frings GmbH) filled with 4.5 L under strictly anaerobic conditions. An 80:20 H_2_:CO_2_ gas mixture was continuously supplied at a flow rate of 2.5 L min^−1^ and a constant overpressure of 700 mbar. The bioreactor was equipped with sensors and control systems for H_2_ safety, pH, temperature, and agitation, and was maintained at 37°C with continuous stirring at 200 rpm to ensure homogeneous mixing. The reactor was then inoculated with 340 mL of *C. ljungdahlii* pre‐cultures, which corresponds to the same inoculation ratio as in the MES reactors. The pH was maintained at 5.7 by the automated addition of 1 M NaOH and 10% H_3_PO_4_. Samples were collected daily for OD_600_ and HPLC measurements, and biomass was collected during the active phase for physiological comparison.

### 2.2 Transmission Electron Microscopy and Fluorescence Labeling

Imaging was conducted using a Zeiss EM900 transmission electron microscope, upgraded with digital capabilities by Point Electronic, and operated at an accelerating voltage of 80 kV.

Cyanophycin was labelled using a rabbit polyclonal antibody to L‐arginine (Antibodies.com, cat. no. A81941), and fluorescence and brightfield images were acquired using a Keyence BZ‐X800 microscope equipped with a 60× Plan‐Apochromat oil immersion objective lens (60×/1.40 Oil WD 0.13). Image‐based quantification was performed in Fiji/ImageJ (Schindelin et al. 2012). For cell length analysis and cyanophycin size, TEM images were calibrated using the scale bar, and the Feret's diameter was used. Cell counts were determined from TEM images in Fiji/ImageJ using the Cell Counter plugin. Cells were manually classified as intact or structurally disrupted based on TEM morphology, and the resulting counts were used for quantitative comparison between conditions.

### 2.3 Protein Extraction and in‐Solution Digestion

Cells were lysed in 1% SDS, 150 mM NaCl, and 100 mM triethylammonium bicarbonate (TEAB) with the addition of 1 tablet each of complete Ultra Protease Inhibitor Cocktail and PhosStop (both from Roche) per 10 mL of lysis buffer. Subsequently, cysteine thiols were reduced and carbamidomethylated in one step for 30 min at 70°C by the addition of 2 μL of 500 mM TCEP (tris(2‐carboxyethyl)phosphine) and 625 mM 2‐chloroacetamide (CAA) per 100 μg of total protein in 100 μL. Proteins were digested for 18 h at 37°C after the addition of Trypsin/Lys‐C mix at a protease‐to‐protein ratio of 1:25. The resulting peptides were prepared for LC–MS/MS analysis. LC–MS/MS method and data analysis method are in SI.

### 2.4 RNA Isolation and Library Preparation

RNA was extracted from *C. ljungdahlii* DSMZ 13528 of OD_600_ ~ 0.3 from the gas fermenter, and OD_600_ ~ 0.12–0.16 from MES reactors. Cells were collected in tubes containing RNAlater solution, and RNA was isolated using the Quick‐RNA Miniprep Kit (Zymo Research, USA). Transcriptome analysis was performed as previously described (PMIDs: 31313835, 39727155), and detailed library preparation is in the SI. The number of usable RNA‐seq samples was limited by the low biomass and cell integrity obtained under MES conditions. Because *C. ljungdahlii* produced substantially less biomass during MES than during GF, the recovery of sufficient high‐quality RNA for sequencing was challenging. Only samples meeting the RNA quality threshold required for library preparation were included in the transcriptomic analysis, resulting in two replicates for RNA‐seq.

### 2.5 Transcriptome Library Preparation and Sequencing

Ribosomal RNA depletion, cDNA library preparation, and sequencing were performed as previously described, and details of RNA sequencing, liquid chromatography and tandem mass spectrometry, and bioinformatics analysis are provided in the Supporting Information.

## 3 Results

### 3.1 Gas Fermentation Outperforms Microbial Electrosynthesis Using *C. ljungdahlii* , Whereas Microbial Electrosynthesis Yields a Broader Product Spectrum

**FIGURE 1:**
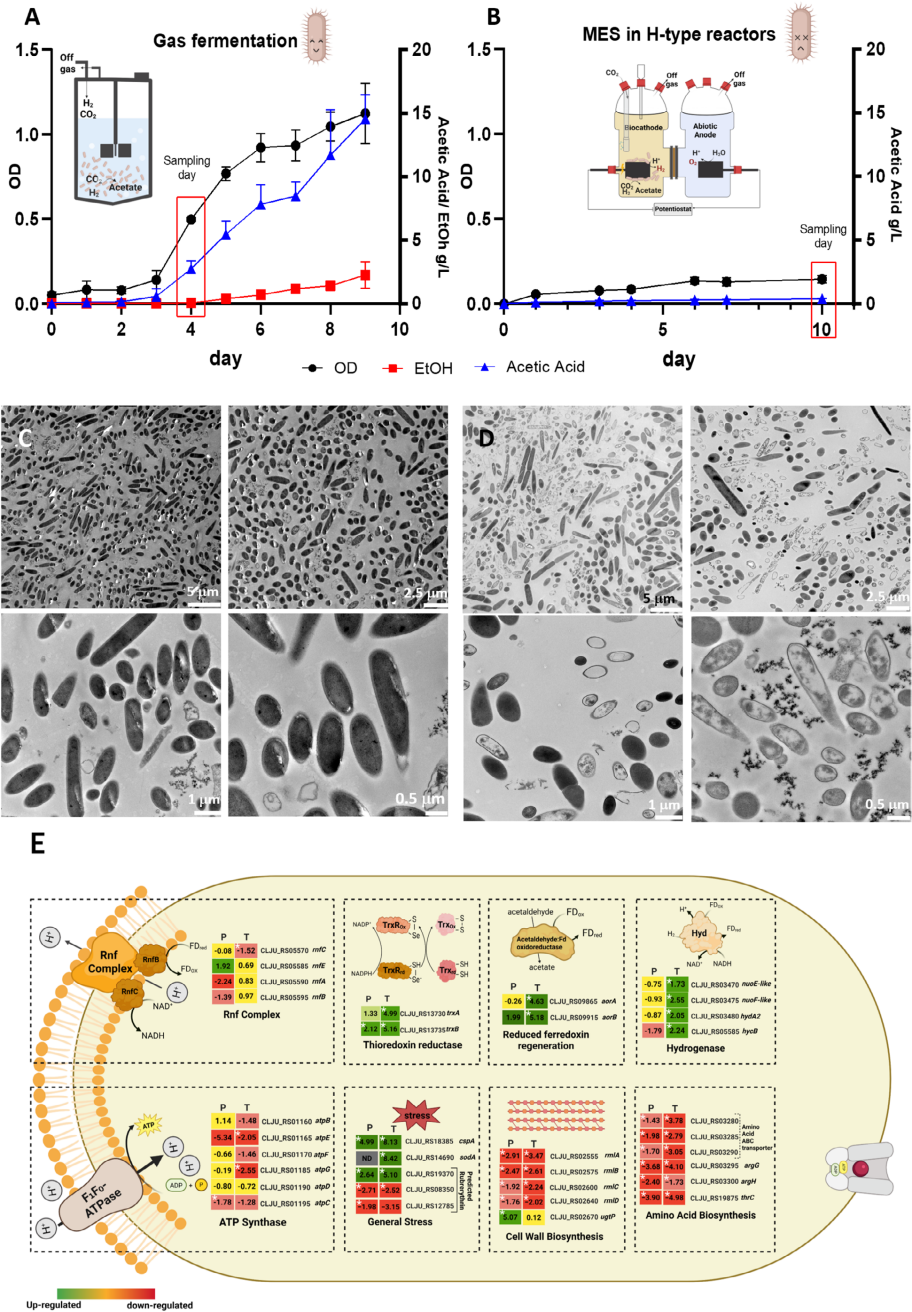
Comparison of microbial growth under GF and MES conditions and change in the redox state compared to GF under MES. (A, B) Bioprocess performance showing optical density (OD_600_), acetate, and ethanol production to be much higher in Gas Fermentation (GF) in (A) compared to (B) Microbial Electrosynthesis (MES). A sketch of the respective process operation is included to highlight similarities and dissimilarities of operation. (C, D) Transmission electron microscopy (TEM) images of cells grown in GF (C), which look more intact than the cells growing in MES (D), showing signs of stress and structural damage. (E) Schematic overview of key changes in redox, stress, and biosynthetic pathways on the proteomic and transcriptomic levels under MES conditions compared to GF. Coloured heatmap panels show log_2_ fold change (P, proteomics, T transcriptome) for selected marker genes. (Bioprocesses both GF and MES *n* = 3), (Proteomics *n* = 3, Transcriptomics *n* = 2), * Asterisks denote significance (*p* < 0.05). Reduced ferredoxin regeneration: (*aorB*, *aorA*) tungsten‐containing aldehyde: Ferredoxin oxidoreductases. Thioredoxin system: (*trxA, trxB*) thioredoxin reductase genes. General stress response: (*cspA*) carbon starvation protein A, (*sodA*) superoxide dismutase. Amino acid biosynthesis: (*argG*) argininosuccinate synthase, (*argH*) argininosuccinate lyase, (*thrC*) threonine synthase; Hydrogenase and electron transfer: (*nuoE/F*‐*like*), (*hydA2*), (*hycB*) hydrogenases and associated electron transfer proteins. Cell wall biosynthesis/dTDP‐L‐rhamnose pathway: (*rmlA*, *rmlB*, *rmlC*, *rmlD*) core enzymes of dTDP‐L‐rhamnose biosynthesis, (*ugtP*) glycosyltransferase family 4 (*gt4*).

To understand the limitations of MES, we compared the performance of *C. ljungdahlii* for GF and MES in terms of optical density (OD_600_) and product titers. Both processes were inoculated with the same cell‐to‐media volume ratio. At the end of the MES run, the mean OD_600_ of *C. ljungdahlii* was 0.145. By comparison, GF reached a mean OD_600_ of 1.124, corresponding to an 8‐fold difference (Figure 1A,B). Likewise, the mean acetate concentration in MES was 0.560 g L^−1^, whereas GF reached 11.69 g L^−1^ by Day 8, corresponding to a 21‐fold difference. Ethanol was detected only during GF, likely because it was flushed out during MES by the continuous gas flow of CO_2_/N_2_. On the other hand, two amino compounds, ethanolamine and glycine, were produced only in MES, with maximum concentrations of 22.4 and 14.7 mg L^−1^, respectively (Table S1). Transmission Electron Microscopy (TEM) imaging of biomass samples from both bioprocesses provided an initial indication that cells exhibit different morphologies and signs of disintegration, suggesting they may be in a distinct physiological state during MES operation (Figure 1C,D). Cells from GF appeared intact and at the usual cell size (1–3 μm) (Tanner et al. 1993). While the cells from MES were smaller, many were either empty or showed signs of compromised membrane integrity, indicating a stressed environment in H‐type reactors. Quantification of apparent cell length from TEM images showed that cells under MES conditions were significantly smaller than in the control condition. Across the MES datasets, the mean apparent cell length was 0.97 μm, compared with 1.75 μm in GF samples, corresponding to a 1.8‐fold difference (Welch's *t*‐test, *p* = 7.52 × 10^−13^). Moreover, the analysis of cell integrity from TEM images showed a significant difference between conditions (*n* = 156 cells per condition). MES samples contained a substantially higher proportion of structurally disrupted cells than the GF samples (54.5% vs. 17.9%; chi‐square test, *p* = 4.22 × 10^−11^), indicating compromised cellular integrity under MES conditions.

We performed transcriptomic and proteomic analyses to investigate molecular‐level physiological changes and differences in *C. ljungdahlii* during the two autotrophic bioprocesses (Figure S1A–F), detecting 3181 and 2019 active genes, respectively (Tables S2 and S3). Of these, 178 genes were differentially expressed in the transcriptomic dataset (*p* ≤ 0.05, log_2_FC ≥ 2 or ≤ −2) (Figure S2A), and 162 in the proteomic dataset, with 58 genes overlapping between the two datasets. The results showed that under MES, *C. ljungdahlii* shifts its energy metabolism by downregulating biosynthetic pathways and upregulating alternative ATP‐generating routes (Figure 1E). Gene Ontology (GO) enrichment analysis highlighted significant changes in the Wood–Ljungdahl pathway (WLP), as well as pathways related to amino acid and carbohydrate metabolism (Figure S2B–D). Pathways downregulated during growth in MES are primarily involved in energy‐intensive biosynthetic processes, including purine nucleotide, ribonucleotide, organophosphate, and organonitrogen biosynthesis, indicating energy depletion and metabolic stress during MESSI (Figure S2C,D). In contrast, upregulated pathways during growth in MES are associated with energy generation and metabolic adaptation, including ATP regeneration, cell adhesion and motility, and arginine catabolism. To further elucidate metabolic adaptations, we examined clusters of differentially expressed genes co‐localized on the genome by plotting the log_2_FC of each gene versus its genomic location and examining operons that show significant changes. For detailed pathway information, see Supporting Information: Results Section 1 and Figures S1 and S2. These results are context‐dependent and should be interpreted in light of the specific MES configuration and operating conditions used here.

### 3.2 General Stress Response and Major Changes in Energy Conservation Reflect the Severe Physiological Stress During Microbial Electrosynthesis

In addition to the TEM images showing severe cell damage, we identified multiple differentially expressed stress‐associated genes. Notably, the carbon starvation protein A was among the top upregulated genes (Figure 1E, Table S4). Genes involved in oxidative stress defence show mixed regulation during MES, suggesting a broader redox stress rather than a response to oxygen exposure; for details, see Supporting Information: Results Section 1. Another sign of redox stress is the strong induction of the thioredoxin and glutathione systems (Figure 1E), which are known to maintain cellular redox homeostasis (Lu and Holmgren 2014; Lushchak 2012).

Beyond redox stress, the observed severe cellular damage suggests more disruptions in energy homeostasis. Our omics analysis revealed downregulation of several ATP synthase subunit genes, most notably *atpE*, encoding ATP synthase subunit c, consistent with reduced ATP synthase assembly or activity during MES (Figure 1E). The obvious energetic consequence is the noted reduced growth and performance during MES, despite a highly reduced intracellular environment. In *C. ljungdahlii* , Δp for ATP synthesis is created by a proton gradient generated by the Rnf complex, a membrane‐bound ferredoxin:NAD^+^ oxidoreductase that couples electron transfer to outward proton translocation (Figure 1E). As suggested by our previous study (Boto et al. 2023), cells may experience a high NADH/NAD+ ratio during MES, and accordingly show increased expression of NADH‐consuming reactions, including the alcohol/aldehyde dehydrogenase (CLJU_RS08100) and acetaldehyde dehydrogenase (CLJU_RS05870), while maintaining pathways that may contribute to regeneration of the reduced ferredoxin required to establish Δp. Our data show upregulation of two tungsten‐containing aldehyde:ferredoxin oxidoreductase genes (Figure 1E), which are known to maintain the reduced ferredoxin pool. In addition, the low growth observed under MES was accompanied by broad repression of cell division and biosynthetic genes (For details, see Supporting Information: Results Section 1).

### 3.3 Major Changes in the Wood‐Ljungdahl Pathway and the Diversion of the Methyl Branch Toward Glycine Under the Electrochemical Environment

**FIGURE 2:**
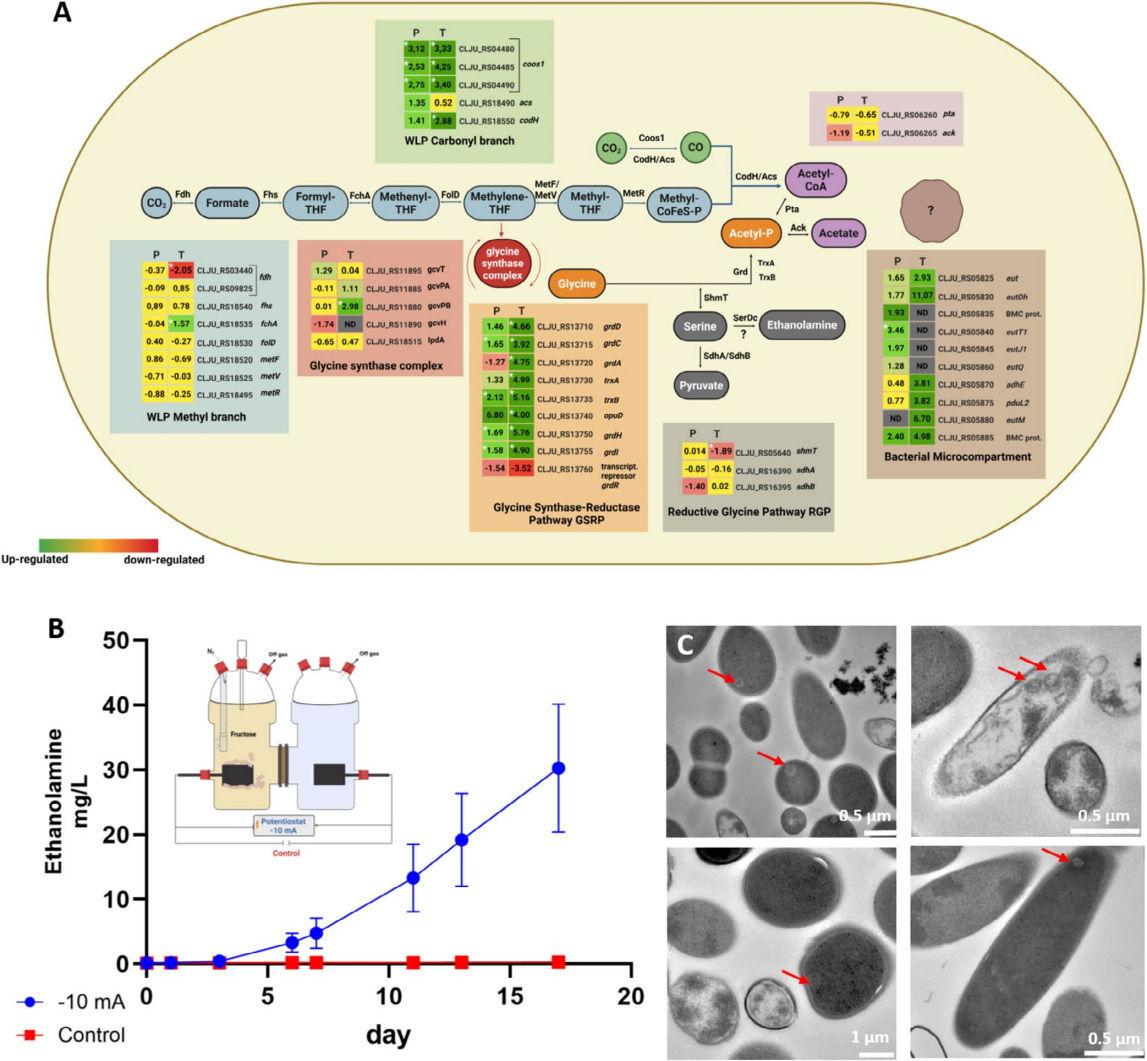
MES‐driven changes of the Wood‐Ljungdahl Pathway (WLP) and related pathways. (A) Coloured heatmap panels show log_2_ fold change (P, proteomics T, transcriptome) for selected marker genes in WLP and GSRP (follow colour‐code to match expression data to pathway element). Under MES, the carbonyl branch of the WLP is upregulated, enhancing CO_2_ fixation. In contrast, *formate dehydrogenase* in the methyl branch is downregulated, likely due to the production of abiotic formate at the cathode. The glycine synthase‐reductase pathway (GSRP) is strongly upregulated at both the transcriptional and translational levels. BMC‐associated genes show strong upregulation, though the specific reactions compartmentalized therein remain unidentified. (Proteomics *n* = 3, Transcriptomics *n* = 2). * Asterisks denote significance (*p* < 0.05). (B) Ethanolamine is only produced with the current applied to the reactors, but not in controls, although the starvation effect was excluded by fructose supplementation (*n* = 3). (C) Distinct structures resembling bacterial microcompartments were observed during MES. Carbonyl branch: (*cooS1*) carbon monoxide dehydrogenase subunit, (*acd*) acetaldehyde dehydrogenase, (*codh*) carbon monoxide dehydrogenase, (*pta*) phosphotransacetylase, (ack) acetate kinase. Methyl branch: (*fdh*) formate dehydrogenase, (fhs) formate‐tetrahydrofolate ligase, (*fchA*) formyltetrahydrofolate cyclohydrolase, (*folD*) Bifunctional protein, (*metF*) and (*metV*) methylene tetrahydrofolate reductase, (*meTr*) methyltetrahydrofolate: Corrinoid/Fe‐S protein methyltransferase. Glycine synthase complex: (*gcvT*) aminomethyltransferase, (*gcvPA*) glycine dehydrogenase subunit P, (*gcvPB*) glycine dehydrogenase subunit P, (*gcvH*) glycine cleavage system H protein, (*lpdA*) dihydrolipoamide dehydrogenase. GSRP: (*grdD*) glycine reductase subunit D, (*grdC*) glycine reductase subunit C, (*grdA*) glycine reductase subunit A, (*trxA*) thioredoxin, (*trxB*) thioredoxin reductase, (*opuD*) glycine betaine/carnitine/choline transporter, (*grdH*) glycine reductase subunit H, (*grdI*) glycine reductase subunit I, (*grdR*) glycine reductase transcriptional regulator. RGP: (*shmT*) serine hydroxymethyltransferase, (*sdhA*) L‐serine dehydratase, (*sdhB*) L‐serine deaminase. BMC: (*eut*) ethanolamine utilization protein, (*eutDH*) ethanolamine dehydrogenase, (BMC prot) microcompartment protein, (*eutT1*) ethanolamine cobalamin adenosyltransferase, (*eutJ1*) chaperone protein DnaK, (*eutQ*) ethanolamine utilization protein EutQ, (*adhE*) acetaldehyde/ethanol dehydrogenase, (*pduL2*) phosphate propanoyltransferase, (*eutM*) ethanolamine utilization microcompartment protein.

The Wood‐Ljungdahl Pathway (WLP) is a central pathway for carbon fixation in acetogens. The WLP consists of a carbonyl (Eastern) branch, where no ATP input is required, and a methyl (Western) branch, which consumes one ATP. Our omics investigations indicate increased expression of the carbonyl branch (Figure 2A), whereas expression of the methyl branch remained largely unchanged. One exception was the transcriptional downregulation of the *fdhA gene* (CLJU_RS03440), which encodes formate dehydrogenase. Formate can also be produced abiotically by electrochemical reduction of CO_2_ at the cathode (Boto et al. 2023). The downregulation of fdhA may indicate a direct use of formate, bypassing the metabolic reduction step from CO_2_ to formate.

An alternative ATP‐generating pathway closely linked to the WLP is the glycine synthase‐reductase pathway (GSRP), which we found upregulated during MES (Figure 2A, Figure S3A). GSRP activation aligns with the exclusive production of glycine and ethanolamine during MES (Table S1) (Boto et al. 2023; Kuchenbuch et al. 2025). GSRP shares the first four reactions with the WLP methyl branch, converting CO_2_ to methylene‐THF (Figure 2A, Song et al. 2020), which is then used, along with NADH and ammonia, for glycine synthesis. Glycine can be further processed via either the GSRP or the reductive glycine pathway (RGP) to yield acetyl‐phosphate or serine, respectively, which would open a direct route to ethanolamine synthesis (Song et al. 2020). While genes comprising GSRP were upregulated during MES, the RGP pathway was not (Figure 2A), suggesting that *C. ljungdahlii* may redirect carbon flux toward ethanolamine through alternative routes. For detailed pathway information, see Supporting Information: Results Section 2.

We next asked whether activation of the GSRP and the formation of ethanolamine are directly linked to the electrochemical environment. To test this, we cultivated *C. ljungdahlii* in an H‐type reactor, supplementing it with fructose for heterotrophic growth to eliminate carbon or energy starvation. Three of the six MES reactors were operated electrochemically with a galvanostatic current of −10 mA (resulting in a cathode potential of around −900 mV); the other three remained unaffected by electrochemistry (open‐circuit control). Although cells under both conditions looked healthy and intact in TEM evaluation (Figure S4), ethanolamine was observed only when electric current was applied, confirming that its production is linked to the electrochemical environment rather than starvation or general stress response (Figure 2B).

### 3.4 Upregulation of Bacterial Microcompartment Genes During MES Suggests a Role in Processing Toxic Intermediates

Comparative genomic studies have previously revealed that the *C. ljungdahlii* genome contains genes for Bacterial Microcompartments (BMCs) distributed across two locations with high similarity. BMCs are commonly used to shield the cell from toxic intermediates. (Kerfeld et al. 2018). Previously, BMCs were found to be upregulated during heterotrophic growth of *C. ljungdahlii* compared to autotrophic growth, suggesting that BMCs host the reaction of methylglyoxal produced from glycolysis into 1‐propanol (Aklujkar et al. 2017). In our omics analysis, we detected a strong upregulation of one of the two BMC gene loci during MES (Figure 2A, Figure S3B, Table S5; for details, see Supporting Information: Results Section 2).

Beyond the genetic evidence, the actual presence of BMCs in *C. ljungdahlii* has not been reported before. In TEM images of the MES‐derived cells, we observed the frequent presence of cellular compartments of similar size and structure to known BMCs, which were always located near the cell envelope (Figure 2C). The previous suggestion that BMCs in *C. ljungdahlii* host the reaction of glycolytic methylglyoxal detoxification seems unfitting here, as glycolysis is inactive under our growth conditions. Although several glycolysis/gluconeogenesis enzymes appear to be upregulated in KEGG pathway analysis (Figure S2C), these correspond to the gluconeogenic direction of carbon flow, as the MES condition lacks external sugars and relies on CO_2_ fixation, thereby reversing flux through shared enzymes. However, methylglyoxal is also derived from the metabolism of glycine (Lai et al. 2022), which aligns with our observation of GSRP activation. Another possibility is that BMCs sequester toxic intermediates from ethanolamine utilization. Ethanolamine breaks down into ammonia and acetaldehyde; ammonia is utilized as a nitrogen source, while acetaldehyde is a toxic intermediate that is converted inside BMCs into acetyl‐phosphate and acetyl‐CoA (Khatri et al. 2012; Wade et al. 2019).

### 3.5 The Arginine Deaminase Pathway (ADI) is Upregulated During MES and May Support ATP Generation Under Stress

**FIGURE 3:**
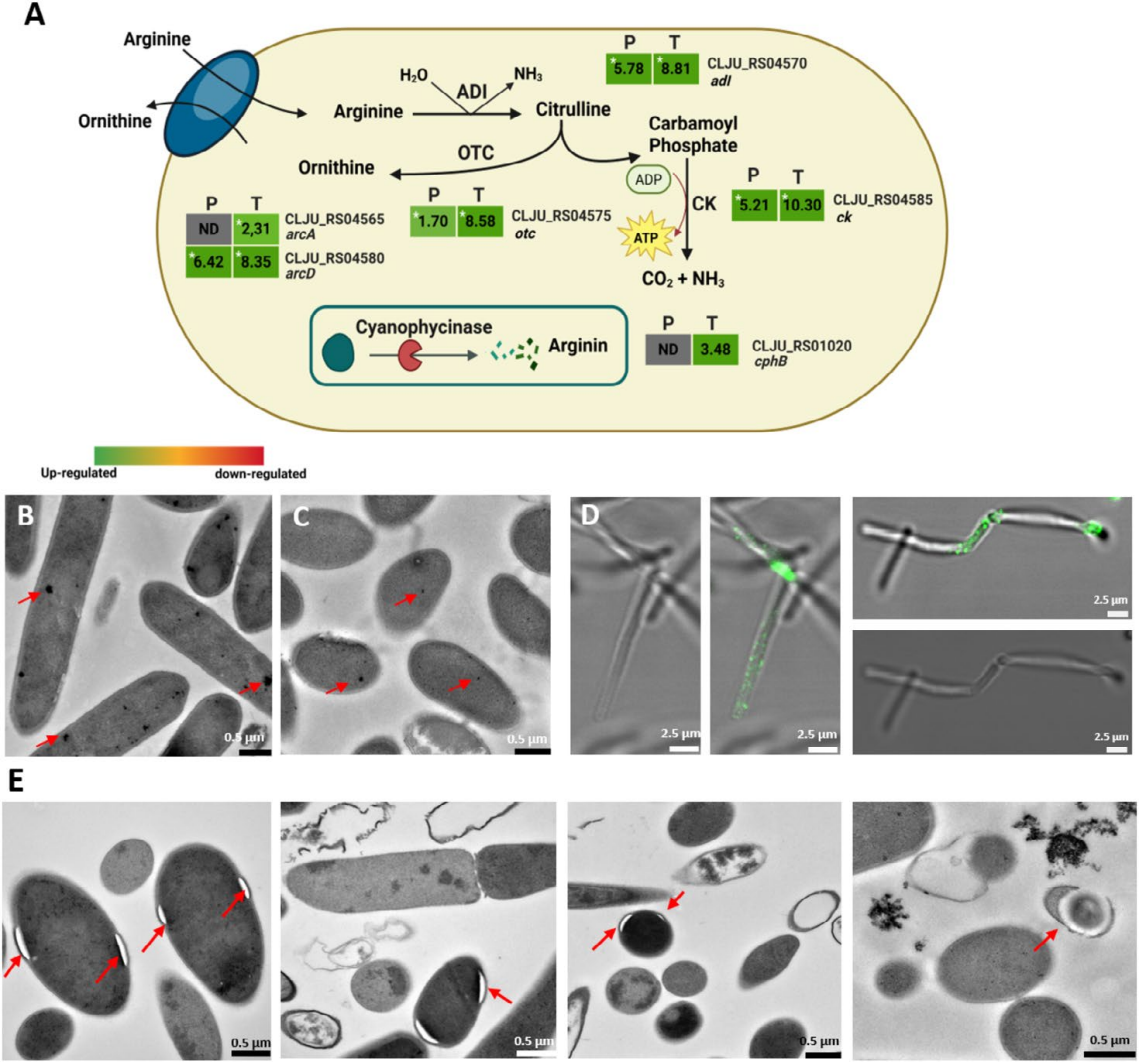
Upregulation of the Arginine Deaminase Pathway (ADI) and cyanophycin degradation under MES conditions. (A) Coloured heatmap panels show log_2_FC (P, proteomics T, transcriptome) for the ADI pathway that is activated under MES. Four key ADI pathway genes are upregulated at both RNA and protein levels: Arginine/ornithine antiporter, ornithine carbamoyltransferase, carbamate kinase, and arginine deiminase. Cyanophycinase is also upregulated at the transcriptomic level, suggesting that cyanophycin breakdown may supply arginine to fuel the ADI pathway, thereby supporting ATP generation. (Proteomics *n* = 3, Transcriptomics *n* = 2). * Asterisks denote significance (*p* < 0.05). (B) TEM images of *C. ljungdahlii* growing autotrophically in GF, showing abundant and larger potential cyanophycin granules compared to (C) under MES, where putative cyanophycin granules in cells appear smaller. (D) Immunolabelling of cyanophycin with anti‐L‐arginine antibodies in cells of *C. ljungdahlii* growing heterotrophically (see controls in Figure S5). (E) Cells from MES showing white gaps near the cell wall, indicating cytoplasmic membrane contraction associated with early sporulation or cell death. Arginine deaminase pathway: (*arcA*) arginine: ornithine antiporter, (*arcD*) arginine: ornithine antiporter, (*adi*) arginine deiminase, (*otc*) ornithine carbamoyltransferase, (*ck*) carbamate kinase, (*cphB*) cyanophycinase.

Under MES conditions, four genes encoding the Arginine Deaminase (ADI) pathway were upregulated (Figure 3A, Figure S3C). ADI is known to be activated in response to stress as an alternative ATP‐generating pathway (Díez et al. 2017; Gallego et al. 2012; Valgepea et al. 2017). A possible arginine source for ADI is the storage polymer cyanophycin. The cyanophycinase gene (*cphB*), which depolymerizes cyanophycins explicitly, was highly expressed in our transcriptomic data (Figure 3A). In our TEM images, we observed dense black granules morphologically identical to cyanophycin inclusions previously described in cyanobacteria (Liang et al. 2014). Quantitative analysis of cyanophycin granule diameter from TEM images showed that granules under MES conditions were significantly smaller than in GF (Figure 3B,C). Across the pooled datasets, 34 granules were analysed per condition. The mean granule diameter under MES was 0.018 μm (±0.014 μm), compared with 0.073 μm (±0.043 μm) in the control, corresponding to a 4.0‐fold difference that was highly significant (Welch's *t*‐test, *p* = 1.75 × 10^−8^). Aligning with our multi‐omics data and indicating active cyanophycin degradation during MES. To confirm cyanophycin formation, we performed fluorescent immunolabelling with anti‐L‐arginine antibodies, which showed the presence of aggregations being labelled therewith (Figure 3D, Figure S5A–F). Repeated attempts to extract cyanophycin were unsuccessful, which we attribute to the low cell density, very low levels of the polymer (see Figure S5A–F, compared to cyanobacteria), or that they are tightly bound to cellular structures. Previous studies have linked cyanophycins with sporulation (Liu et al. 2016), but under MES conditions, we observed that the gene for the master sporulation regulator, Spo0A, was downregulated. In contrast, more than 15 sporulation‐associated genes were upregulated (Figure S3D), suggesting that in *C. ljungdahlii* , *Spo0A* may not act as the only sporulation regulator and that alternative pathways contribute to spore formation. Additionally, the location of *cphB* within these upregulated sporulation‐related genes strengthens the suggestion that it may play a role in sporulation, along with the observation of cells with a white gap near the cell wall in TEM images, which is indicative of early‐stage spore development (Figure 3E). Re‐cultivating C. *ljungdahlii* after 24 h of heat treatment at 80°C resulted in growth, supporting the likely formation of spores. However, direct functional validation remains challenging in *C. ljungdahlii* because genetic manipulation in this organism is still difficult.

## 4 Discussion

Despite its promise as a sustainable CCU technology, microbial electrosynthesis from CO_2_ remains poorly understood at the level of microbial metabolism and physiology of pure cultures. For over a decade, efforts to improve MES have primarily relied on engineering solutions and mixed cultures, treating the biocatalyst as a black box. (Harnisch et al. 2024). This lack of physiological understanding may limit further advancement of MES toward application.

**FIGURE 4:**
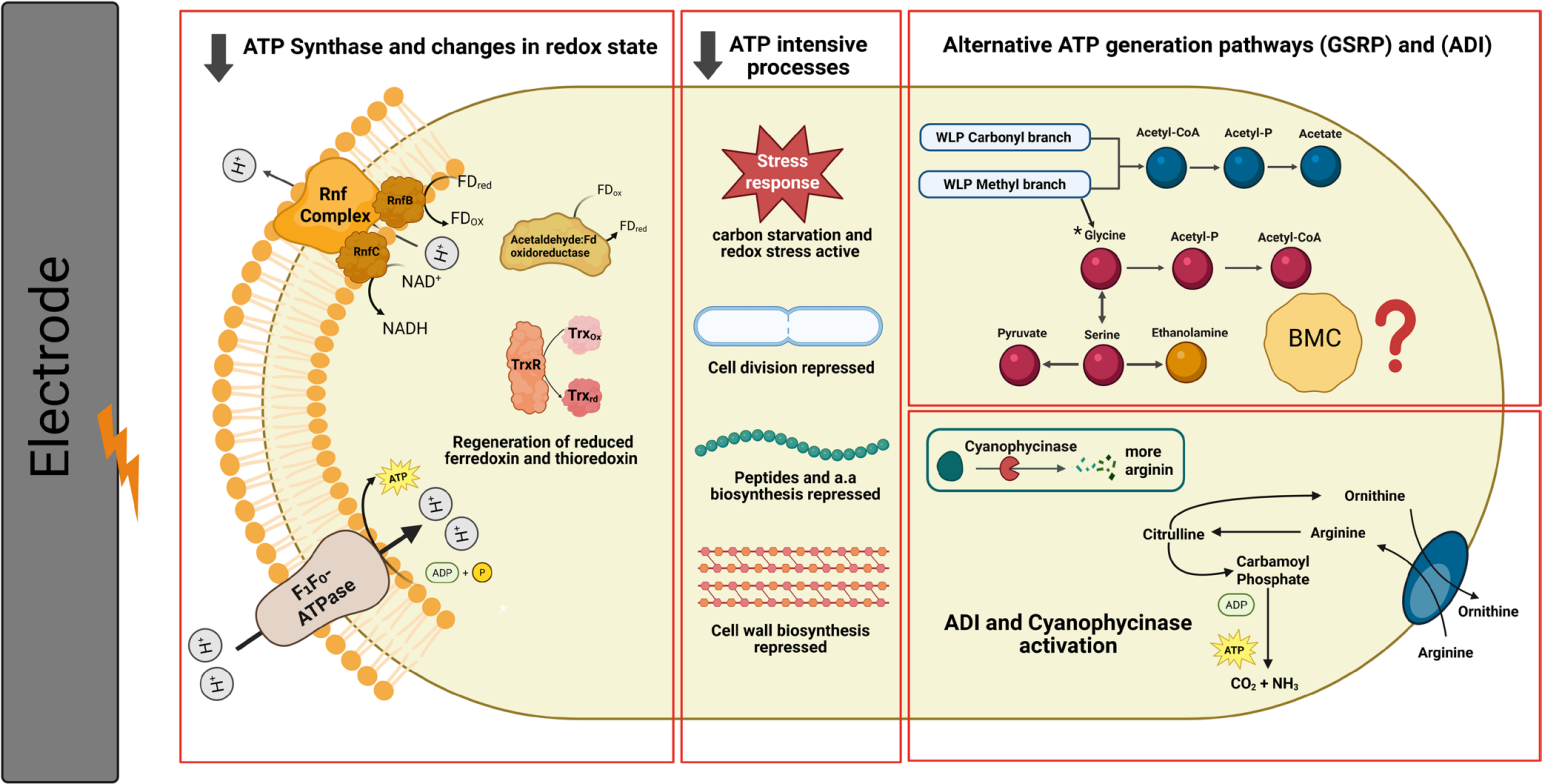
Summary of the main physiological responses in *Clostridium ljungdahlii* under MES.

In this study, we investigated the physiological responses of the model acetogen *Clostridium ljungdahlii* during MES in H‐type reactors fed with CO_2_ and in situ electrolytic H_2_, compared to classical gas fermentation with CO_2_ and H_2_ as substrates, to identify physiological constraints associated with MES. Since *C. ljungdahlii* in MES and GF are fed the same carbon and energy source, it could be presumed that the cells in both processes share a similar metabolism and physiology. However, our findings show that the MES setup strongly affects the biocatalytic performance (Figure 4) and indicate that the electrochemical environment, whose effect on microbes is largely understudied, substantially alters microbial metabolism and fitness. These findings should, however, be interpreted in the context of the specific operational conditions used here, including reactor architecture, gas supply, hydrogen availability, and electrochemical operation. TEM imaging further revealed that cellular integrity was severely compromised during MES, with many cells exhibiting signs of death and fragmentation. Even when carbon and energy limitations were minimized by heterotrophic growth in an electrochemical environment, we still observed shifts in metabolic reactions, as ethanolamine was produced exclusively in the electrochemical condition. Besides the strong upregulation of multiple stress response strategies (e.g., carbon starvation protein, oxidative stress factors), the results indicate that ATP synthesis via the proton motive force (Δp) was negatively affected, and ATP‐consuming pathways were strongly downregulated, including those involved in cell wall, amino acid, peptide, and nucleotide biosynthesis. At first glance, this energy starvation could be explained by the electron donor, that is, H_2_ limitation.

However, the increased expression of alternative electron transfer systems, such as those involving NADH, ferredoxin, and the thioredoxin network, points to a highly reduced intracellular state and enrichment of redox‐active cofactors. This highly reduced cellular state may lead to the activation of pathways, such as the Glycine Synthase‐Reductase Pathway (GSRP), which has been linked to the elevated NADPH levels based on a genome‐scale model (Boto et al. 2023). Since GSRP co‐utilizes the first four steps of the methyl branch to convert CO_2_ to methylene‐THF (Song et al. 2020), diversion of the methyl branch toward glycine may contribute to the imbalance between the two WLP branches. Our results indicate the direct formate feed to the methyl branch, consistent with our previous work, which showed the abiotic production of formate on the cathode (Boto et al. 2023). Our results also showed that this activation is linked to the electrochemical setup rather than a starvation‐induced effect of MES. Moreover, while Boto et al. found that during MES, *C. ljungdahlii* biofilms had lower growth and acetate yields, glycine and ethanolamine production were higher than in planktonic cells. This is consistent with our results, which suggest that closer exposure to the negatively poised cathode could lead to a more reduced cellular state. Despite the close connection between the GSRP and RGP pathways, the exact pathway by which *C. ljungdahlii* produces ethanolamine could not be conclusively resolved, as we could not detect the gene for serine decarboxylase, which links glycine to ethanolamine synthesis (Figure 4).

Cells face a highly reduced intracellular state and impaired ATP synthase efficiency under MES, leading to ATP starvation. To conserve energy, cells suppress ATP‐intensive processes, including biosynthesis and cell division. Major changes occur in the Wood‐Ljungdahl Pathway (WLP), with the methyl branch diverted toward glycine metabolism and the induction of a bacterial microcompartment (BMC). The arginine deaminase pathway (ADI) is activated as an alternative ATP‐yielding route, likely fueled by cyanophycin degradation.

Despite the highly reduced state under MES, our data suggest that Δp may be disrupted, potentially through increased proton permeability or through electrochemical effects on proton distribution at the cell envelope. Maintaining redox homeostasis is essential for activity and proliferation (Strahl and Hamoen 2010). Thus, impaired ATP synthesis due to membrane depolarization may create an energy limitation that underlies the stress responses detected here. Consistent with this energetic limitation, *C. ljungdahlii* showed increased expression of alternative ATP‐generating pathways, including the glycine reduction pathway and the arginine deiminase pathway (Figure 4). Our transcriptomic data further suggest that cyanophycin contributes to fueling the ADI pathway. The possible native production of cyanophycin in a gas‐fermentation model organism, such as *C. ljungdahlii* , would be a promising alternative approach to producing this valuable biopolymer, which has potential applications as biomedical materials, fertilizers, and biodegradable plastics (Kwiatos and Steinbüchel 2021). However, despite repeated attempts, we were unable to extract cyanophycin from *C. ljungdahlii* , possibly due to its low abundance or its tight association with cellular structures. For a more detailed discussion of cyanophycins, we refer to the Supporting Information: discussion Section S1.

Another relevant change during MES is the induction of BMCs. Although BMCs have been described already in several genomic and experimental studies of *C. ljungdahlii* , the predicted function of those compartments varied entirely across those works, and currently, no reliable hypothesis can be derived for their function (see Supporting Information: discussion Section S2 for a discussion of those studies). We initiated deeper investigations to determine the exact function of those BMCs and hope to clarify their role in future work.

While our study strongly advances the current understanding of the physiological response during MES, it also raises a critical question: How does the applied negative cathode potential (in this work −0.9 V vs. Ag/AgCl) and the resulting electrochemical environment affect the intracellular redox balance, membrane energetics, and hence cellular fitness? It remains unclear whether the severe stress observed, both in our physiological analysis and in image analysis, is due to direct physical contact of *C. ljungdahlii* with the electrode surface or can be attributed to exposure to an electric field and ion migration, including electroosmosis, that affects the redox environment. Maintaining redox homeostasis is essential for the activity and proliferation of cells (Benyamin et al. 2023; Strahl and Hamoen 2010; Stratford et al. 2019) and thus hampering ATP‐synthesis by membrane depolarization creates energy limitation and could be responsible for the here detected stress response. Exposure to an electric field and creating ion imbalances across the membrane can lead to depolarization or at least a lower membrane potential as required by the ATP‐synthase for ATP‐generation (Tremblay et al. 2013). Thereby, this effect is especially pronounced for prokaryotes, where displacement of a few hundred ions can change the membrane potential by several mV (Benarroch and Asally 2020). This stress effect also depends on the distance to the electrode surface based on the Debye length of the electrostatic force. This is in line with our previous work showing that biofilms exhibited higher stress levels, although interestingly, viable cells were still found directly on the electrode surface, suggesting that direct contact with the cathode does not entirely kill the cells (Boto et al. 2023). Thus, simply isolating the electrode or shielding the cells to avoid immediate physical contact may not be sufficient to mitigate MES‐related stress. Instead, a deeper understanding is needed of how the electrochemical environment harms the redox homeostasis of the cells; this includes assessing whether a certain distance from the cathode could provide sufficient stress relief. An important open question is whether the observed physiological response to MES conditions is specific to *C. ljungdahlii* or represents a general phenomenon among acetogens and other model organisms used in microbial electrosynthesis. Similar physiological screening of diverse MES model organisms could reveal variability in tolerance to electrochemical conditions. Such differences may reflect adaptations in membrane composition or in energy‐conservation mechanisms. Identifying suitable strains for MES conditions would help improve MES performance and reliability. In this context, two strategies can be used: (i) systematic screening of existing strains to identify better‐suited biocatalysts, and (ii) the development of adapted strains through adaptive laboratory evolution under MES conditions. What is becoming clear is that, in its current state, MES pushes *C. ljungdahlii* into a distinct physiological state in which novel pathways are activated, different from the classical lithotrophic growth conditions. These findings suggest that MES should be considered not only as a promising technology for CCU and electricity storage from renewable sources, but also as a valuable platform for studying microbial adaptation. These findings now set the stage for more focused metabolic analysis during MES, which can guide strain as well as reactor system engineering and, importantly, adaptive laboratory evolution to develop strains that can withstand the highly reducing environment imposed by MES.

## Author Contributions

**Falk Harnisch:** conceptualization, supervision, funding acquisition, writing – review and editing. **Martin Westermann:** resources, investigation, formal analysis, writing – review and editing. **Sara Al Sbei:** conceptualization, methodology, data curation, formal analysis, investigation, writing – original draft, writing – review and editing, visualization. **Miriam A. Rosenbaum:** conceptualization, methodology, funding acquisition, project administration, supervision, writing – review and editing. **Kai Papenfort:** conceptualization, resources, writing – review and editing. **Aurélie Jost:** resources, investigation, writing – review and editing. **Thomas Krüger:** methodology, investigation, writing – review and editing. **Axel A. Brakhage:** resources, writing – review and editing. **Santiago T. Boto:** conceptualization, methodology, supervision, writing – review and editing.

## Funding

This work was supported by Deutsche Forschungsgemeinschaft, SPP2240 e‐Biotech, project no. 445388719, CRC Transregio 124 ‘FungiNet’, project ID 210879364, EXC 2051 – Project number 390713860, 460889961, EXC 2051–Project‐ID 390713860. European Research Council, CoG‐864669, CoG‐101088027.

## Conflicts of Interest

The authors declare no conflicts of interest.

## Supporting information


**Table S1:** MES and GF metabolites and aminocompounds.

**Table S2:** RNA seq data of all detected genes given as log_2_FoldChange MES versus GF.

**Table S3:** Proteomics data of all detected proteins given as log_2_Ratio MES versus GF.

**Table S4:** Omics data for specific genes/proteins related to redox stress.

**Table S5:** Omics data for specific genes/proteins related to the main affected metabolic pathways.

**Figure S1:** Integrated transcriptomic and proteomic analysis of *Clostridium ljungdahlii* for MES vs GF (A, B) Volcano plots showing the distribution of differentially expressed genes (A, transcriptomics) and proteins (B, proteomics). Red dashed lines indicate thresholds for significance. (C, D) Mean–difference (MA) plots displaying log_2_ fold change versus mean expression for RNA‐Seq (C) and proteomics (D). (E, F) Genomic position plots showing log_2_ fold change values across their genomic start positions, showing the co‐localization of DEG related to the same pathway.
**Figure S2:** Overview of the omics analysis of *Clostridium ljungdahlii* for MES vs GF (A) Venn diagram showing the overlap between differentially expressed genes (transcriptome, blue) and proteins (proteome, red), with 58 shared features. (B) The top differentially expressed genes were identified in both datasets. Bars represent RNA (blue) and protein (red) levels. (C) KEGG module enrichment analysis showing significantly enriched pathways based on integrated transcriptomic and proteomic data. Modules involved in central carbon metabolism exhibit mixed expression, where some genes are upregulated while others are downregulated. In contrast, pathways linked to stress adaptation, motility, and transport systems are upregulated. (D) Gene Ontology (GO) enrichment analysis of differentially expressed pathways common between proteomics and transcriptomics. Pathways related to biosynthesis and ATP consumption are highly downregulated, and genes associated with energy generation, cell motility, and adhesion are upregulated. Bubble size indicates the number of genes per term, and colour indicates adjusted *p*‐values. (*n* = 3 Proteomics, *n* = 2 Transcriptomics).
**Figure S3:** The main affected pathways on both transcriptomic and proteomic level of MES vs GF in *Clostridium ljungdahlii*. (A) Activation of the Glycine‐Serine‐Reductive Pathway (GSRP) under MES conditions, showing consistent upregulation in both transcriptomic and proteomic data during MES, with the exception of *grdA*, which is downregulated at the protein level. The known repressor of GSRP is also downregulated. (B) Expression levels of bacterial microcompartment (BMC)‐associated genes demonstrate coordinated upregulation at both the transcriptional and translational levels under MES conditions. (C) Upregulation of four core genes of the arginine deiminase (ADI) pathway is observed at both the transcriptomic and proteomic levels, accompanied by transcriptional upregulation of cyanophycinase. (D) Genes associated with sporulation show increased expression at the transcriptomic level, but not at the proteomic level.
**Figure S4:** Transmission Electron Microscopy (TEM) of *C. ljungdahlii* growing heterotrophically with and without applied current. With fructose supplementation, cells look healthy, although metabolic changes were detected to be induced by the electrochemical environment.
**Figure S5:** Microscopic images for cyanophycin labeling with fluorescent anti‐L‐arginine antibodies. (A and B) *C. ljungdahlii* under heterotrophic conditions. (C and D) represent the same labeling in *Cyanobacterium synechocystis* sp. strain PCC 6803 used as a positive control. (E and F) Labeling with *Escherichia coli* as a negative control. The same amount of antibodies was applied to all samples, and imaging was recorded with the same settings.

## Data Availability

All transmission electron microscopy (TEM) and fluorescence immunolabelling image datasets generated in this study have been deposited in the BioImage Archive (https://www.ebi.ac.uk/bioimage‐archive/) under accession number TMP_2025‐11‐17T10:52:33.688358143Z. The mass spectrometry proteomics data have been deposited in the ProteomeXchange Consortium via the PRIDE (Perez‐Riverol et al., 2025) partner repository, with the dataset identifier PXD070356 and 10.6019/PXD070356. Raw sequence data for this RNA‐Seq experiment are archived in the European Nucleotide Archive (ENA), with direct links provided via the ArrayExpress record under accession number ArrayExpress accession identifier E‐MTAB‐16180. Processed data and associated metadata are available for download from ArrayExpress, following MINSEQE standards.
