# Supplementary material for: Microbial Electrosynthesis Reshapes Energy Metabolism and Physiology in Clostridium ljungdahlii
Source: Microb Biotechnol. 2026 Jun 6;19(6):e70398. doi: 10.1111/1751-7915.70398 (PMC13241827; doi:10.1111/1751-7915.70398)
Supplement: Supplementary file 6 — Figure S1: Integrated transcriptomic and proteomic analysis of Clostridium ljungdahlii for MES vs GF (A, B) Volcano plots showing the distribution of differentially expressed genes (A, transcriptomics) and proteins (B, proteomics). Red dashed lines indicate thresholds for significance. (C, D) Mean–difference (MA) plots displaying log2 fold change versus mean expression for RNA‐Seq (C) and proteomics (D). (E, F) Genomic position plots showing log2 fold change values across their genomic start positions, showing the co‐localization of DEG related to the same pathway. Figure S2: Overview of the omics analysis of Clostridium ljungdahlii for MES vs GF (A) Venn diagram showing the overlap between differentially expressed genes (transcriptome, blue) and proteins (proteome, red), with 58 shared features. (B) The top differentially expressed genes were identified in both datasets. Bars represent RNA (blue) and protein (red) levels. (C) KEGG module enrichment analysis showing significantly enriched pathways based on integrated transcriptomic and proteomic data. Modules involved in central carbon metabolism exhibit mixed expression, where some genes are upregulated while others are downregulated. In contrast, pathways linked to stress adaptation, motility, and transport systems are upregulated. (D) Gene Ontology (GO) enrichment analysis of differentially expressed pathways common between proteomics and transcriptomics. Pathways related to biosynthesis and ATP consumption are highly downregulated, and genes associated with energy generation, cell motility, and adhesion are upregulated. Bubble size indicates the number of genes per term, and colour indicates adjusted p‐values. (n = 3 Proteomics, n = 2 Transcriptomics). Figure S3: The main affected pathways on both transcriptomic and proteomic level of MES vs GF in Clostridium ljungdahlii. (A) Activation of the Glycine‐Serine‐Reductive Pathway (GSRP) under MES conditions, showing consistent upregulation in both transcriptomic and [file MBT2-19-e70398-s004.docx]

**Supporting Information for**

**Microbial electrosynthesis reshapes energy metabolism and physiology in *Clostridium ljungdahlii***

**Sara Al Sbei ^1,2^, Santiago T. Boto ^1,2^, Thomas Krüger^3^, Kai Papenfort ^2,4^, Martin Westermann ^5^, Aurelie Jost ^6,4^, Falk Harnisch ^7^, Axel A. Brakhage ^2,3,4^, Miriam A. Rosenbaum ^1,2,4 *^**

^1^ Bio Pilot Plant, Leibniz Institute for Natural Product Research and Infection Biology – Hans-Knöll-Institute, Jena, Germany

^2^ Institute of Microbiology, Faculty of Biological Sciences, Friedrich Schiller University Jena, Germany

^3^ Molecular and Applied Microbiology, Leibniz Institute for Natural Product Research and Infection Biology – Hans-Knöll-Institute, Jena, Germany

^4^ Cluster of Excellence Balance of the Microverse, Friedrich-Schiller-University Jena, Jena, Germany

^5^ Electron Microscopy Center, Jena University Hospital, Jena, Germany

^6^ Microverse Imaging Center, Friedrich-Schiller-University Jena, Germany

^7^ Environmental Microbiology, UFZ-Helmholtz Centre for Environmental Research, Leipzig, Germany

* Correspondence to: [Miriam.rosenbaum@leibniz-hki.de](mailto:Miriam.rosenbaum@leibniz-hki.de)

ORCID: 0000-0002-4566-8624

**Table of Contents:**

| **Section** | **Extended Data Title** | **Page** |
| --- | --- | --- |
| **Result S1** | **Overview of the omics analysis of *Clostridium ljungdahlii* for MES vs GF** | 3 |
| Figure S1 | Integrated transcriptomic and proteomic analysis of *Clostridium ljungdahlii* for MES vs GF | 5 |
| Figure S2 | Overview of the omics analysis of *Clostridium ljungdahlii* for MES vs GF | 6 |
| **Result S2** | **Activation of the Glycine-Serine-Reductive Pathway (GSRP) under microbial electrosynthesis** | 8 |
| **Result S3** | **Expression of bacterial microcompartment (BMC) genes under microbial electrosynthesis** | 8 |
| Figure S3 | The main affected pathways on both the transcriptomic and proteomic levels of MES vs GF in *Clostridium ljungdahlii* | 10 |
| Figure S4 | Transmission Electron Microscopy (TEM) of *C. ljungdahlii* growing heterotrophically with and without applied current | 11 |
| **Discussion S1** | **The role of cyanophycins in *C. ljungdahlii*** | 12 |
| Figure S5 | Microscopic images for cyanophycin labeling with fluorescent anti-L-arginine antibodies | 13 |
| **Discussion S2** | **The role of Bacterial Microcompartments (BMCs) in *C. ljungdahlii*** | 14 |
| **SI Methods** | **SI Materials and Methods** | 15 |
| SI References | SI References | 20 |
|  | **Extended Supplementary Information Tables (Excel Document)** |  |
| Table S1 | Aminocompounds and Metabolites of MES and GF | Sheet 1 |
| Table S2 | Transcriptomics dataset | Sheet 2 |
| Table S3 | Proteomics dataset | Sheet 3 |
| Table S4 | Redox-related genes | Sheet 4 |
| Table S5 | Affected pathways | Sheet 5 |

**SI Results:**

**S1. Overview of the omics analysis of *Clostridium ljungdahlii* for MES vs GF**

- *This supplementary section contains information on the overall comparative omics analysis of* C. ljungdahlii *in our work and some detailed results on the physiological state that are important but secondary to the main results presented in results chapters 1 and 2 of the main text*

We performed transcriptomic and proteomic analyses to investigate molecular-level physiological changes and differences of *C. ljungdahlii* during the two autotrophic bioprocesses, detecting 3,181 and 2,019 active genes, respectively (SI Table S2 and S3). Of these, 178 genes were differentially expressed in the transcriptomic dataset (p ≤ 0.05, log₂FC ≥ 2 or ≤ −2) (SI Fig. S1), and 162 in the proteomic dataset, with 58 genes overlapping between the two datasets (SI Fig. S2). Gene Ontology (GO) and KEGG module enrichment analysis revealed significant changes in the Wood–Ljungdahl pathway (WLP), as well as in biosynthetic pathways related to cofactor, amino acid, and carbohydrate metabolism (SI Fig. S2). Pathways downregulated during growth in MES are primarily involved in energy-intensive biosynthetic processes, including purine nucleotide, ribonucleotide, organophosphate, and organonitrogen biosynthesis, which highlights the energy depletion and metabolic stress experienced during MES (Fig. 1E Main Text). In contrast, upregulated pathways during growth in MES are associated with energy generation and metabolic adaptation, including alternative ATP generation pathways that are independent of the use of the proton motive force. To further elucidate metabolic adaptations, we examined, on the one hand, known stress response mechanisms to understand the observed physiological distress in *C. ljungdahlii* during MES. On the other hand, we evaluated clusters of differentially expressed genes co-localized on the genome by plotting the log₂ fold change (log₂FC) of each gene against its genomic location and examining clusters that show significant changes (SI Fig. S1E and F).

The reduced growth observed under MES conditions indicates impaired cell proliferation and our omics analysis shows that cells reprogram their energy metabolism by downregulating biosynthetic pathways and upregulating stress response and alternative ATP-generating routes (Fig. 1E Main Text, SI Tables S4). In more detail, transcriptomic data showed significant downregulation of key cell division genes, including *whiA* (a DNA-binding regulator initiating cell division), the minCDE system (ensuring correct septum placement), and*ftsQ* (an essential

divisome component facilitating septal assembly). Also, processes for biosynthesis of cofactors, amino acids, and cell wall components were broadly repressed, likely to minimize ATP usage. Cells strongly downregulated amino acid ABC transporters, and histidine biosynthesis was suppressed by downregulating the rate‑limiting enzyme (CLJU_RS05720), ATP phosphoribosyltransferase. A cluster of genes (CLJU_RS02555–RS02640) encoding the dTDP-L-rhamnose biosynthesis pathway showed coordinated transcriptional and translational downregulation. However, a single glycosyltransferase family 4 (GT4) protein (CLJU_RS02670) was highly expressed, as shown by proteomics, in MES (log₂FC = +5.07, p = 0.0009). GT4 enzymes are known to be involved in cell envelope modifications (Martinez-Fleites et al., 2006) suggesting that CLJU_RS02670 may mediate surface charge remodeling to survive the highly reduced MES conditions. In addition, several genes associated with oxidative stress defense were differentially expressed in MES. The rubrerythrin gene (CLJU_RS19370), previously identified as a key peroxide scavenger under oxidative challenge in *C. ljungdahlii* (Whitham et al., 2015), was highly upregulated along with the superoxide dismutase (SOD, CLJU_RS14690), which detoxifies superoxide radicals. However, other rubrerythrins, such as CLJU_RS08350 and CLJU_RS12785, were downregulated, indicating that these mixed changes in oxidative stress-related genes point toward a general redox stress rather than oxygen exposure. Research on *Mycobacterium* has shown that uncontrolled reductive stress may lead to the formation of radical oxygen species (ROS) through enzymatic shunt reactions, creating a mixed redox challenge for the cells (Mavi et al., 2020).

**
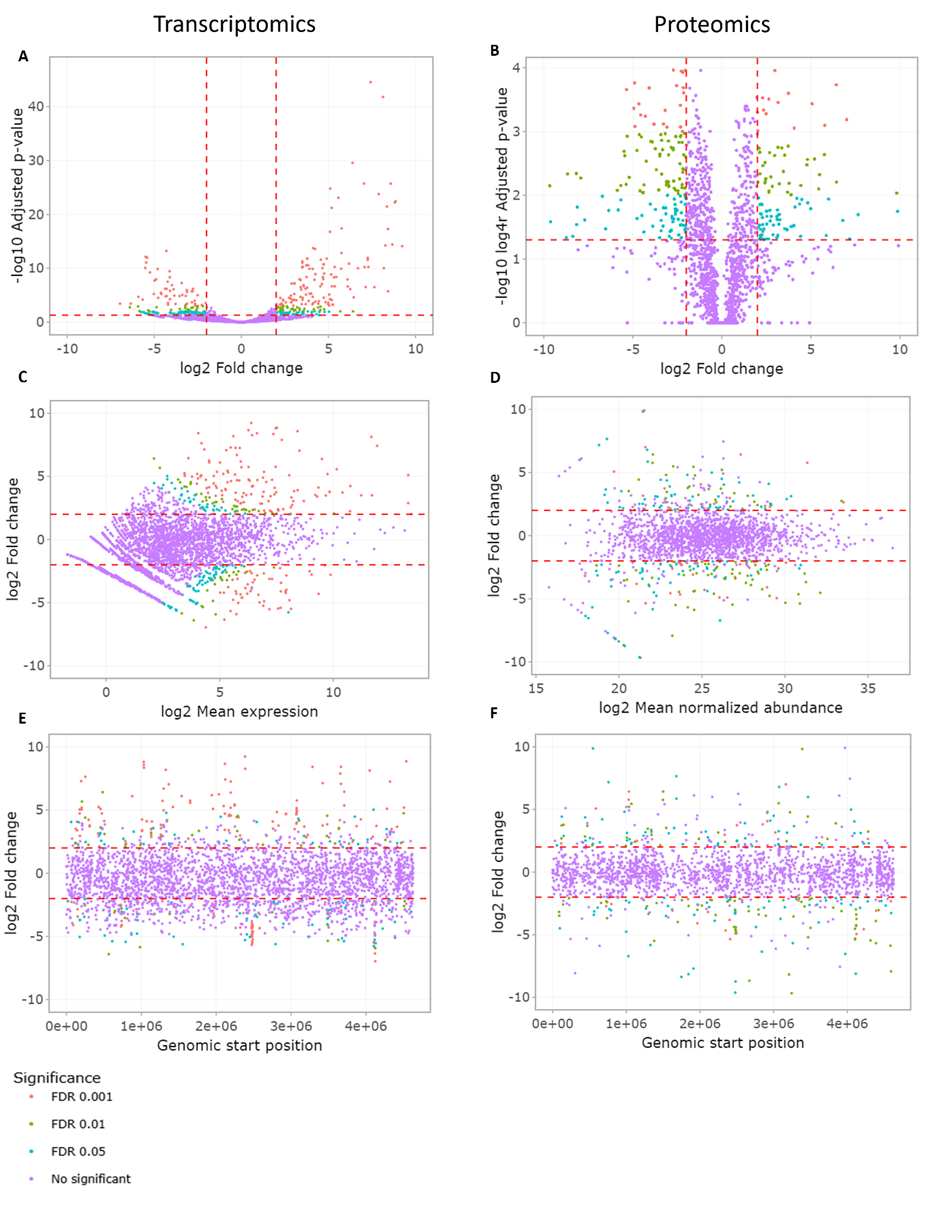
**

**Fig S1: Integrated transcriptomic and proteomic analysis of *Clostridium ljungdahlii* for MES vs GF** (A, B) Volcano plots showing the distribution of differentially expressed genes (A, transcriptomics) and proteins (B, proteomics). Red dashed lines indicate thresholds for significance. (C, D) Mean–difference (MA) plots displaying log_2_ fold change versus mean expression for RNA-Seq (C) and proteomics (D). (E, F) Genomic position plots showing log_2_ fold change values across their genomic start positions, showing the co-localization of DEG related to the same pathway.

Data points are color-coded by significance thresholds: FDR 0.001 (red), FDR 0.01 (green), FDR 0.05 (blue), and non-significant (purple). (n=3 Proteomics, n = 2 Transcriptomics)

**
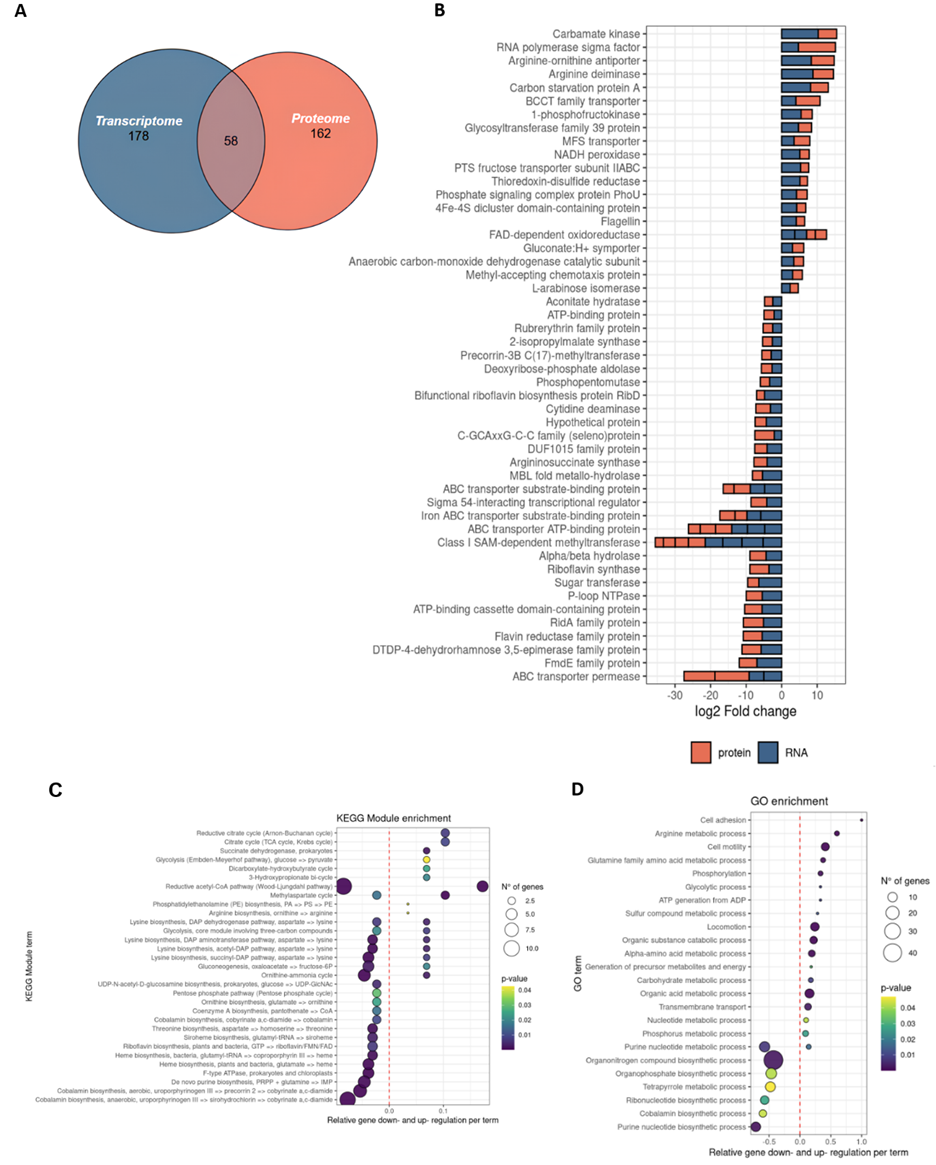
**

**Fig. S2: Overview of the omics analysis of *Clostridium ljungdahlii* for MES vs GF** (A) Venn diagram showing the overlap between differentially expressed genes (transcriptome, blue) and proteins (proteome, red), with 58 shared features. (B) The top differentially expressed genes were identified in both datasets. Bars represent RNA (blue) and protein (red) levels. (C) KEGG module enrichment analysis showing significantly enriched pathways based on integrated transcriptomic and proteomic data. Modules involved in central carbon metabolism exhibit mixed expression, where some genes are upregulated while others are downregulated. In contrast, pathways linked to stress adaptation, motility, and transport systems are upregulated. (D) Gene Ontology (GO) enrichment analysis of differentially expressed pathways common between proteomics and transcriptomics. Pathways related to biosynthesis and ATP consumption are highly downregulated, and genes associated with energy generation, cell motility, and adhesion are upregulated. Bubble size indicates the number of genes per term, and color indicates adjusted p-values. (n=3 Proteomics, n = 2 Transcriptomics).

**S2. Activation of the Glycine-Serine-Reductive Pathway (GSRP) under microbial electrosynthesis**

- *This supplementary section contains more detailed information on the activation of the GSRP as presented in results chapter 3 of the main text*

All genes of GSRP were upregulated in the transcriptomic data, while the putative repressor gene (*grdR*) was downregulated (SI Fig. S3A). Downstream of glycine production, the gene *SHMT* of the RGP, encoding serine hydroxymethyltransferase, is responsible for a bidirectional reaction from glycine to serine. Unexpectedly, *SHMT* was downregulated in the transcriptomic dataset, suggesting that *C. ljungdahlii* may redirect carbon flux toward ethanolamine through alternative routes. Also, the gene encoding serine decarboxylase was not detected in the *C. ljungdahlii* genome.

Taken together, the downregulation of ATP synthase and the upregulation of pathways dependent on alternative cofactors such as NADH, thioredoxin, and ferredoxin suggest that under MES conditions, *C. ljungdahlii* experiences a highly reduced intracellular state. To relieve this redox pressure and compensate for the loss of ATP synthase-mediated energy conservation, the cells appear to activate alternative pathways, such as GSRP, for electron disposal.

**S3. Expression of bacterial microcompartment (BMC) genes under microbial electrosynthesis**

- *This supplementary section contains more detailed information on upregulation of one BMC operon presented in results chapter 4 of the main text*

In our omics analysis, we detected a strong upregulation of the respective gene locus during MES. The entire BMC operon consists of 17 genes (CLJU_RS05805 to CLJU_RS05885) (SI Fig. S3B). Ten genes showed upregulation in the transcriptomic data during MES, while seven were undetected. In the proteomic analysis, ten genes were also found to be upregulated; however, five were not detected. Two were expressed during MES without significant changes compared to GF. Combining both approaches, only the two shortest genes remained undetected (SI Fig. S3B). While some genes in the BMC operon were annotated as propanediol utilization *(Pdu)* genes, others were annotated as ethanolamine utilization *(Eut)* genes, and the primary enzyme was described as a glycyl radical enzyme. However, these inconsistent annotations are based solely on sequence similarity to other species, as there is still no experimental validation of the functions of the enzymes in the BMC locus of *C. ljungdahlii*. Bioinformatic analysis of the BMC operon in *C. ljungdahlii* reveals a chimeric GRM1/GRM3 locus, indicating that the genome has undergone duplication and recombination events (Köpke et al., 2010).


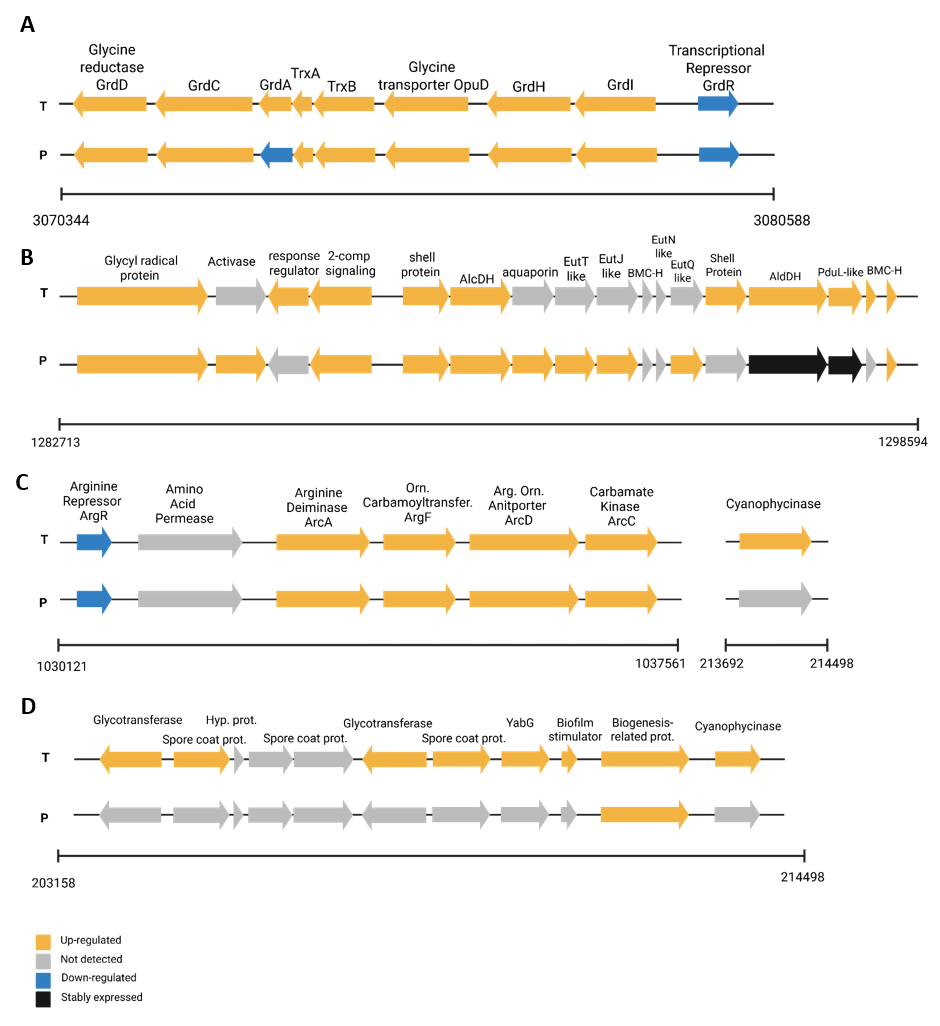


**Fig. S3: The main affected pathways on both transcriptomic and proteomic level of MES vs GF in *Clostridium ljungdahlii.*** (A) Activation of the Glycine-Serine-Reductive Pathway (GSRP) under MES conditions, showing consistent upregulation in both transcriptomic and proteomic data during MES, with the exception of *grdA*, which is downregulated at the protein level. The known repressor of GSRP is also downregulated. (B) Expression levels of bacterial microcompartment (BMC)-associated genes demonstrate coordinated upregulation at both the transcriptional and translational levels under MES conditions. (C) Upregulation of four core genes of the arginine deiminase (ADI) pathway is observed at both the transcriptomic and proteomic levels, accompanied by transcriptional upregulation of cyanophycinase. (D) Genes associated with sporulation show increased expression at the transcriptomic level, but not at the proteomic level.

**
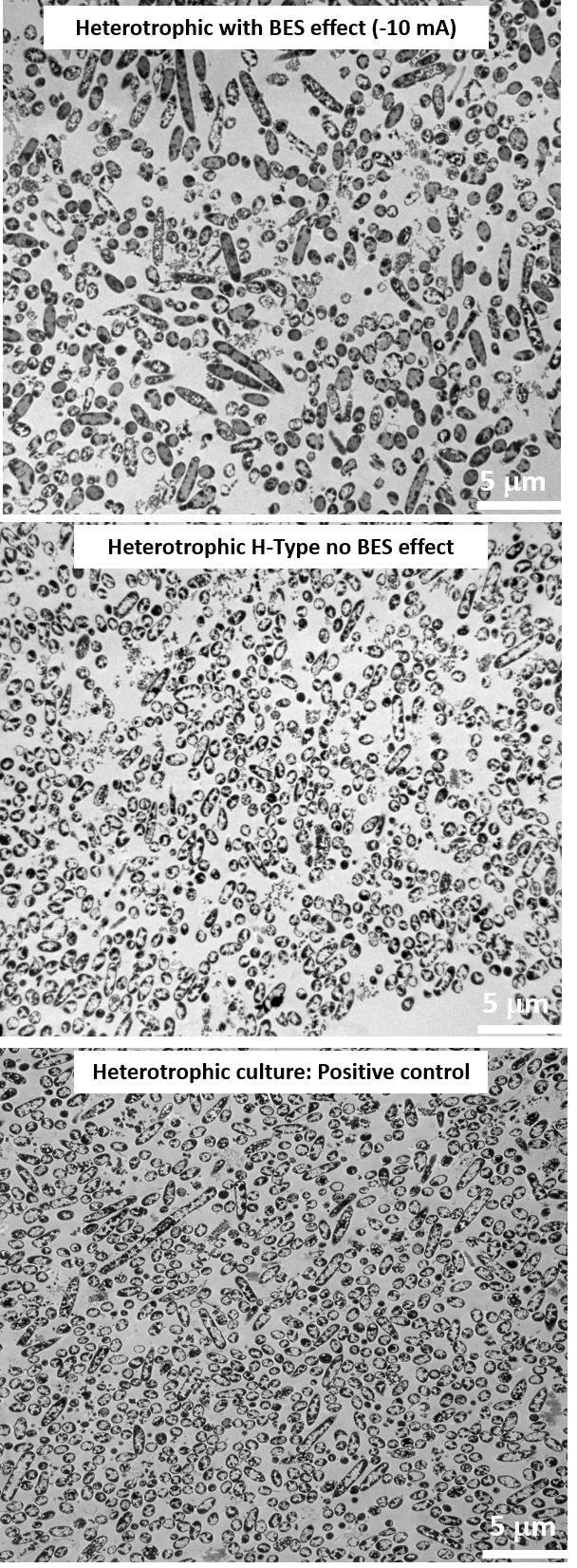
**

**Fig. S4: Transmission Electron Microscopy (TEM) of *C. ljungdahlii* growing heterotrophically with and without applied current.** With fructose supplementation, cells look healthy, although metabolic changes were detected to be induced by the electrochemical environment

**SI Discussion**

**S1. The role of Cyanophycins in *C. ljungdahlii*:**

Cyanophycin was first discovered in cyanobacteria, and numerous attempts have been made to express it in *E. coli* (Frey et al., 2002) as this storage compound has several potential applications.

Although *C. ljungdahlii* possesses the genes for cyanophycin formation (*cphA*) and degradation (*cphB*), only *cphB* activity was detected in our omics data (SI Fig. 3C). Also, previous studies detected *cphB* expression in *C. ljungdahlii*; it was shown to be upregulated in autotrophic growth compared to heterotrophic growth (Aklujkar et al., 2017). However, their work could not confirm whether it acts directly on cyanophycin storage compounds or free (polymerized) arginine from yeast extract.

The localization of *cphAB* genes surrounded by sporulation-related genes in *C. ljungdahlii* may support the observation of Liu et al. for *C. perfringens.* There*,* cyanophycin was implicated in spore formation, and *cphA* disruption led to fewer, smaller spores, despite the polymer being difficult to isolate (Liu et al., 2016). These observations suggest that cyanophycin may also contribute to spore formation in *C. ljungdahlii*, possibly as a structural component, a nitrogen or energy storage, or as part of a broader stress-adaptive program. Alternatively, the genomic co-localization may reflect shared regulatory control during energy-limiting or pre-sporulation conditions. This later hypothesis would better align with our observations in TEM and fluorescence imaging, where we observe multiple free granular structures distributed throughout the cell, which appear smaller in the energy-limited MES cultures than in GF. We attempted correlative imaging of structures observed in TEM images with gold nanobody labeling. However, this failed due to the high imaging contrast of cyanophycins, which overlap with the gold signals. Instead, independent fluorescent antibody labeling of the cells showed a similar distribution of granular structures in cells that were permeable to the antibody. The low abundance of cyanophycins compared to the cyanobacterial control (SI Fig. S5) may explain the inability to extract the granules with known protocols.


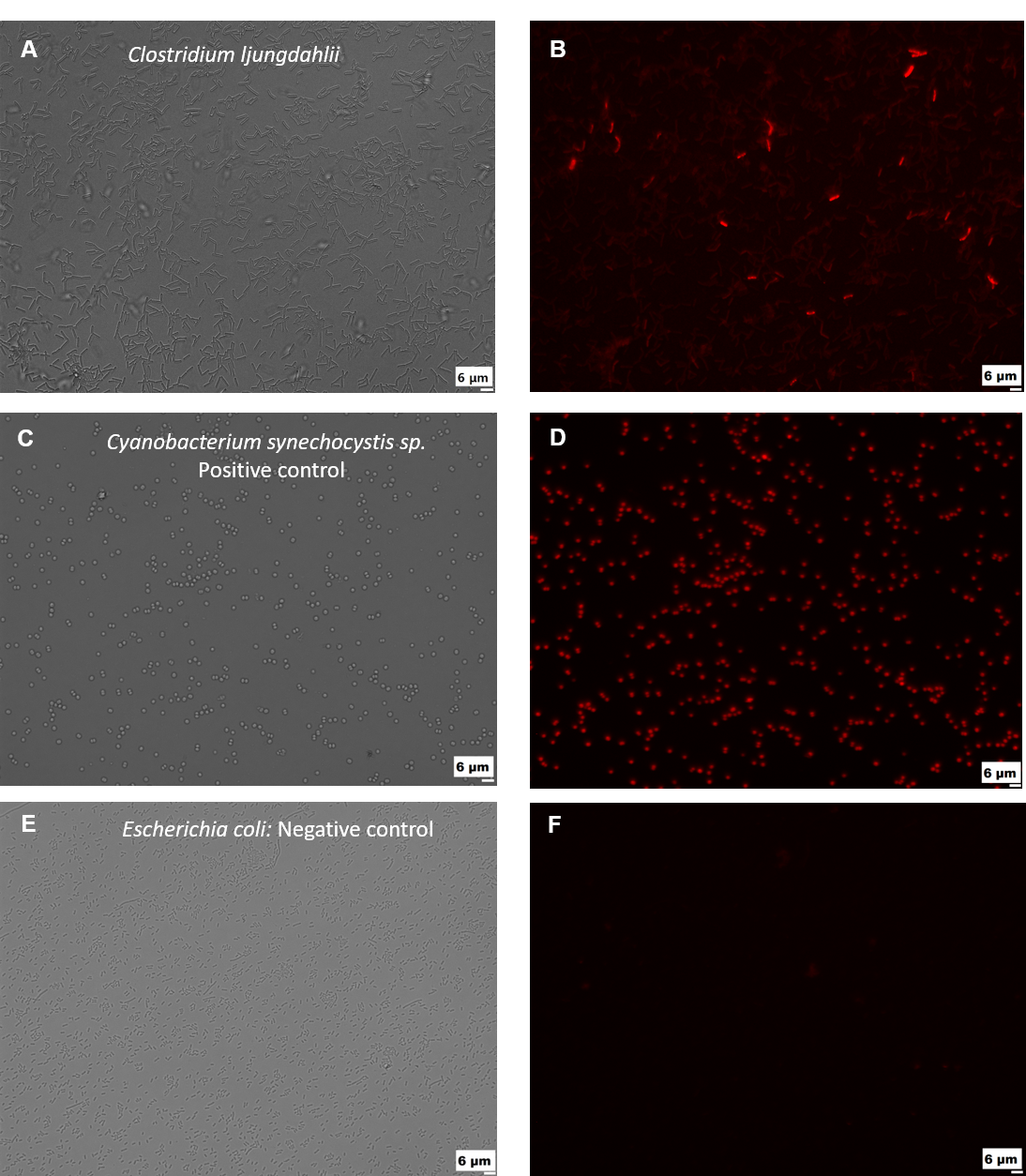


**Fig. S5: Microscopic images for cyanophycin labeling with fluorescent anti-L-arginine antibodies**. (A) and (B) *C. ljungdahlii* under heterotrophic conditions. (C) and (D) represent the same labeling in *Cyanobacterium synechocystis sp.* strain PCC 6803 used as a positive control. (E) and (F) Labeling with *Escherichia coli* as a negative control. The same amount of antibodies was applied to all samples, and imaging was recorded with the same settings.

**S2. The role of Bacterial Microcompartments (BMCs) in *C. ljungdahlii***

BMCs have been described previously in several genomic and experimental studies of *C. ljungdahlii.* Genome analyses of *C. ljungdahlii* by Köpke et al. highlighted the presence of two operons encoding BMCs and suggested that BMCs host the ethanol oxidation reaction. It is also possible that BMC upregulation reflects ethanolamine utilization; ethanolamine breaks down into ammonia and acetaldehyde; ammonia is utilized as a nitrogen source, while acetaldehyde is a toxic intermediate that is usually converted inside BMCs into acetyl-phosphate and acetyl-CoA (Khatri et al., 2012; Wade et al., 2019). Another study found that the BMC operon is downregulated during autotrophic growth compared to heterotrophic growth, suggesting that BMCs function in removing methylglyoxal, a toxic byproduct of glycolysis that is converted to 1-propanol (Aklujkar et al., 2017). The most recent study predicted that BMCs expression in *C. ljungdahlii* is induced by choline and 1,2-propanediol (1,2-PD) (Zhang et al., 2025). Our own analysis delivered inconsistent annotations for the enzymes included in the BMCs, but this is on sequence similarity to other species. Thus, there is a mix of evidence towards a function that currently cannot really be combined to draw a conclusive hypothesis on the specific toxic reaction compartmentalized inside the BMCs and an experimental clarification is urgently required.

**SI Materials and Methods:**

**Bacterial Strain and Cultivation**
The composition of RCM was as follows: 10 g L⁻¹ peptone, 10 g L⁻¹ meat extract, 3 g L⁻¹ yeast extract, 5 g L⁻¹ NaCl, 5 g L⁻¹ fructose, 1 g L⁻¹ soluble starch, 5 g L⁻¹ sodium acetate trihydrate (Na-acetate·3H₂O), 0.5 g L⁻¹ L-cysteine·HCl, and 1 mg L⁻¹ resazurin. RCM was prepared in 500 mL or 1 L borosilicate Duran glass bottles, briefly boiled in a microwave, sparged with 100% nitrogen (N₂) gas for ten minutes to remove dissolved oxygen, and then aliquoted into Hungate tubes (10 mL per tube) under anaerobic conditions (continuous N2 flushing). The Hungate tubes were then autoclaved. The composition of PETC medium included: 20 g L⁻¹ 2-(N-morpholino) ethanesulfonic acid, 100 mL L⁻¹ salt stock solution (10×), 10 mL L⁻¹ trace element solution (100×), 10 mL L⁻¹ vitamin solution (100×), 1 g L⁻¹ yeast extract, 0.3 g L⁻¹ L-cysteine·HCl, and 1 mg L⁻¹ resazurin. The pH of the final PETC medium was adjusted to 5.7 using KOH. The trace element solution (100×) included 0.2 g L⁻¹ CoCl₂·6H₂O, 0.8 g L⁻¹ (NH₄)₂Fe(SO₄)₂, 1 g L⁻¹ MnSO₄, 2 g L⁻¹ nitrilotriacetic acid, 0.2 g L⁻¹ Na₂MoO₄, 0.02 g L⁻¹ Na₂SeO₄, 0.02 g L⁻¹ Na₂WO₄, and 0.2 g L⁻¹ ZnSO₄. The trace element solution was prepared by dissolving nitrilotriacetic acid in distilled water, adjusting the pH to 6.5 with KOH, adding the minerals of the trace metal solution, and then adjusting the final pH to 7.0.
The salt stock solution (10×) contained 20 g L⁻¹ NH₄Cl, 2 g L⁻¹ KCl, 4 g L⁻¹ MgSO₄·7H₂O, 2 g L⁻¹ NaCl, 2 g L⁻¹ KH₂PO₄, and 0.8 g L⁻¹ CaCl₂·2H₂O. The vitamin solution was prepared separately, sterile-filtered (0.2 μm, polypropylene, Agilent Technologies), stored at 4°C, and added freshly to the PETC medium prior to inoculation. The vitamin solution (100×) contained 2 mg L⁻¹ biotin, 5 mg L⁻¹ pantothenic acid, and 5 mg L⁻¹ thiamine·HCl.

**Setup of Microbial Electrosynthesis Reactors**
All gas connections used oxygen-tight Tygon® tubing (VWR, Radnor, USA) and included 0.2 μm PTFE vent filters (Merck, Darmstadt, Germany) to prevent contamination. Each port was equipped with butyl rubber septa (Reichelt Chemietechnik, Heidelberg, Germany) and sealed using oxygen-tight PTFE tape (Würth, Künzelsau, Germany). All potentials are provided versus Ag/AgCl sat. KCl. Ag/AgClsat KCl reference electrodes were prepared by oxidizing a silver wire using a potentiostat (VSP and SP 200, BioLogic Science Instruments, France) and inserting it into a glass chamber filled with saturated KCl Agar. Cyclic Voltammetry was performed before culture inoculation (from −1 V to +0.2 V), at a scan rate of 1 mV s−1. To test the effect of the electrochemical setup while excluding starvation effects, a control was performed. Therefore, the same setup was used, with the addition of 4 mL L-1 sterile anaerobic fructose 50% (w/v) and flushing with humidified pure N2. The reactors were then operated galvanostatically to maintain a constant current input of -10 mA.

**HPLC Method for the Analysis of Metabolites**

Metabolite analysis was performed using an HPLC (Jasco Corporation, Tokyo, Japan). The samples were centrifuged beforehand, and the supernatant was diluted 1:10 with the mobile phase (0.005 M H_2_SO4) for analysis. A 50-μL sample was used for injection. The HPLC system is equipped with an Aminex HPX-87H ion-exclusion column (300 mm x 7.8 mm, 9 µm; Bio-Rad, Hercules, CA, USA) and a Kromasil 100 C18 precolumn (40 mm x 4 mm, 5 µm; Dr. Maisch GmbH, Ammerbuch-Entringen, Germany). The column temperature was kept at 50 °C, and an isocratic flow rate of 0.5 mL min^-1^ was applied. The autosampler was flushed with water in between samples.

The concentrations of the amino compounds ethanolamine and glycine were determined using a separate HPLC system (Jasco Corporation, Japan). Samples (96 μL) were derivatized by mixing with an equal volume of o-phthaldialdehyde reagent, followed by a 2-minute reaction period. (Boto et al., 2023). Subsequently, one μL of the derivatized sample was injected into the HPLC system, equipped with a UV detector (λ = 338 nm) and a fluorescence detector (excitation at 340 nm, emission at 455 nm). Separation was performed on a Kinetex XB C18 column (100 mm × 2.1 mm ID, 2.6 μm; Phenomenex, USA) combined with a SecurityGuard Ultra Cartridge C18 (2.1 mm ID; Phenomenex, USA) maintained at 25 °C. Gradient elution was carried out using mobile phase A (20 mM potassium phosphate buffer, pH 7.2) and mobile phase B (acetonitrile: methanol, 50:50 v/v) at a flow rate of 0.25 mL min^-1^. Figures showing products over time in MES and GF were created using GraphPad.

**Liquid Chromatography and Tandem Mass Spectrometry**

Each sample was measured in triplicate (3 analytical replicates of each biological replicate). LC-MS/MS analysis was performed on an Ultimate 3000 nano RSLC system connected to a QExactive HF mass spectrometer (both Thermo Fisher Scientific, Waltham, MA, USA). Peptide trapping for 5 min on an Acclaim Pep Map 100 column (2 cm x 75 µm, 3 µm) at 5 µL/min was followed by separation on an analytical Acclaim Pep Map RSLC nano column (50 cm x 75 µm, 2µm). Mobile phase gradient elution of eluent A (0.1% (v/v) formic acid in water) mixed with eluent B (0.1% (v/v) formic acid in 90/10 acetonitrile/water) was performed using the following gradient: 0-5 min at 4% B, 30 min at 7% B, 60 min at 10% B, 100 min at 15% B, 140 min at 25% B, 180 min at 45% B, 200 min at 65% B, 210-215 min at 96% B, 215.1-240 min at 4% B. Positively charged ions were generated at spray voltage of 2.2 kV using a stainless steel emitter attached to the Nanospray Flex Ion Source (Thermo Fisher Scientific). The quadrupole/orbitrap instrument was operated in Full MS / data-dependent MS2 mode. Precursor ions were monitored at m/z 300-1500 at a resolution of 120,000 FWHM (full width at half maximum) using a maximum injection time (ITmax) of 120 ms and an AGC (automatic gain control) target of 3×10^6^. Precursor ions with a charge state of z=2-5 were filtered at an isolation width of m/z 4.0 amu for further fragmentation at 28% HCD collision energy. MS2 ions were scanned at 15,000 FWHM (ITmax=100 ms, AGC= 2×10^5^).

**Proteomic Data Processing and Statistical Analysis**

The protein database search was done by searching the tandem mass spectra against the UniProt database (2024/01/31) of *Clostridium ljungdahlii* strain ATCC 55383 (https://www.uniprot.org/proteomes/UP000001656) using Proteome Discoverer (PD) 3.1 (Thermo) and the database search algorithms (threshold search engine scores in parentheses) Chimerys (>2), Mascot 2.8 (>30), Comet (>3), MS Amanda 3.0 (>300), Sequest HT (>3) with and without INFERYS Rescoring. Two missed cleavages were allowed for the tryptic digestion. The precursor mass tolerance was set to 10 ppm, and the fragment mass tolerance was set to 0.02 Da. Modifications were defined as dynamic Met oxidation, phosphorylation of Ser, Thr, and Tyr, protein N-terminal acetylation with or without Met loss, and static Cys carbamidomethylation. A strict false discovery rate (FDR) < 1% (peptide and protein level) was required for positive protein hits. The Percolator node of PD3.1 and a reverse-decoy database were used to validate q-values for spectral matches. Only rank one proteins and peptides of the top-scoring proteins were counted. Label-free protein quantification was based on the Minora algorithm in PD3.1, using precursor abundances determined by intensity and a signal-to-noise ratio greater than 5. Normalization was performed by using the total peptide amount method. Imputation of missing quan values was performed using abundance values corresponding to 75% of the lowest abundance identified per sample. Differential protein abundance was defined as a fold change of >2, pvalue/ABS (log4ratio) <0.05 (pvalue/log4ratio), and at least identified in 2 of 3 replicates of the sample group with the highest abundance. Statistics and data visualization were performed with R 4.3.2 and RStudio 2023.12.0.

**Transcriptome Library Preparation, Sequencing and Data Processing**

Cells were collected in tubes containing RNAlater solution with a 1:4 ratio of RNAlater to sample volume (Invitrogen, USA), mixed gently for 30 seconds, and then centrifuged at 10,000 × g for 15 minutes. Pellets were stored at −80 °C. Later, RNA was isolated using the Quick-RNA Miniprep Kit (Zymo Research, USA), following the manufacturer's protocol with an additional lysis step performed using ZR BashingBead Lysis Tubes containing 0.1 + 0.5 mm beads for 60 seconds in a bead beater (Zymo Research, USA). RNA integrity was assessed using a Bioanalyzer (2100, Agilent; Agilent DNA 7500 Kit). Samples with an RNA integrity number (RIN) of 7 or higher were used. Transcriptome analysis was performed as previously described (PMIDs: 31313835, 39727155). Briefly, ribosomal RNA was depleted using custom-designed biotinylated oligonucleotides as previously described (btn-GACGTGAAGGTAACACCCCTTTCCATACCGAAC) and (btn-GGCAGCGACCTATTCTCCCACAGGGCCTCCCC) (Culviner et al., 2020). Depletion efficiency was verified with an Agilent 2100 Bioanalyzer. cDNA libraries were prepared using the NEBNext Ultra™ II Directional RNA Library Prep Kit for Illumina (NEB, #E7760) following the manufacturer’s protocol. Library quality was evaluated on an Agilent 2100 Bioanalyzer, and sequencing was performed on an Illumina NextSeq 1000/2000 platform.

Raw sequencing reads were evaluated using FastQC (version 0.11.9) for quality, adapter contamination, sequence duplication, and GC content.

Then fastp was used to remove adapters, low-quality bases, and short sequences. The quality of trimmed reads was reassessed using FastQC and summarized using MultiQC (version 1.12). Trimmed reads were aligned to the *Clostridium ljungdahlii* DSM 13528 RefSeq genome (GCF_000143685.1) using Bowtie2. Gene-level read counts were summarized from aligned reads using the tool featureCounts. Differential expression analysis was conducted in R using the DESeq2 package (1.40.2). Gene counts were normalized using the median-of-ratios method to account for differences in sequencing depth and RNA composition. Differential expression between the MES and GF was modeled, using GF condition as the reference. Significance thresholds were based on both fold-change and adjusted p-values (Benjamini-Hochberg corrected), with genes exhibiting an absolute log₂ fold change ≥ 2 and adjusted p-value ≤ 0.05 considered differentially expressed.

Quality control plots, including dispersion estimates and mean–variance relationships, were generated to assess data consistency and the suitability of the statistical model. Annotated gene lists were visualized using volcano and MA plots to display the distribution and the fold changes. Gene ontology (GO) enrichment analyses were performed on the sets of significantly up- and downregulated genes to identify overrepresented biological processes, molecular functions, and cellular components. GO terms were assigned based on annotations derived from the RefSeq GFF files, and statistical significance was determined using Fisher's exact test within the topGO framework. To support functional interpretation, enriched Kyoto Encyclopedia of Genes and Genomes (KEGG) pathways and modules were identified using pathway-based enrichment analysis. The resulting sets of upregulated and downregulated genes were mapped to KO identifiers, and the relative representation within pathways was quantified and compared between experimental conditions.

**References:**

Aklujkar, M., Leang, C., Shrestha, P. M., Shrestha, M., & Lovley, D. R. (2017). Transcriptomic profiles of Clostridium ljungdahlii during lithotrophic growth with syngas or H2 and CO2 compared to organotrophic growth with fructose. *Scientific Reports*, *7*(1). https://doi.org/10.1038/s41598-017-12712-w

Boto, S. T., Bardl, B., Harnisch, F., & Rosenbaum, M. A. (2023). Microbial electrosynthesis with Clostridium ljungdahlii benefits from hydrogen electron mediation and permits a greater variety of products. *Green Chemistry*. https://doi.org/10.1039/d3gc00471f

Culviner, P. H., Guegler, C. K., & Laub, M. T. (2020). A Simple, Cost-Effective, and Robust Method for rRNA Depletion in RNA-Sequencing Studies. *MBio*, *11*(2). https://doi.org/10.1128/mBio.00010-20

Frey, K. M., Oppermann-Sanio, F. B., Schmidt, H., & Steinbüchel, A. (2002). Technical-Scale Production of Cyanophycin with Recombinant Strains of *Escherichia coli*. *Applied and Environmental Microbiology*, *68*(7), 3377–3384. https://doi.org/10.1128/AEM.68.7.3377-3384.2002

Khatri, N., Khatri, I., Subramanian, S., & Raychaudhuri, S. (2012). Ethanolamine utilization in Vibrio alginolyticus. *Biology Direct*, *7*(1), 45. https://doi.org/10.1186/1745-6150-7-45

Köpke, M., Held, C., Hujer, S., Liesegang, H., Wiezer, A., Wollherr, A., Ehrenreich, A., Liebl, W., Gottschalk, G., & Dürre, P. (2010). *Clostridium ljungdahlii* represents a microbial production platform based on syngas. *Proceedings of the National Academy of Sciences*, *107*(29), 13087–13092. https://doi.org/10.1073/pnas.1004716107

Liu, H., Ray, W. K., Helm, R. F., Popham, D. L., & Melville, S. B. (2016). Analysis of the Spore Membrane Proteome in Clostridium perfringens Implicates Cyanophycin in Spore Assembly. *Journal of Bacteriology*, *198*(12), 1773–1782. https://doi.org/10.1128/JB.00212-16

Martinez-Fleites, C., Proctor, M., Roberts, S., Bolam, D. N., Gilbert, H. J., & Davies, G. J. (2006). Insights into the Synthesis of Lipopolysaccharide and Antibiotics through the Structures of Two Retaining Glycosyltransferases from Family GT4. *Chemistry & Biology*, *13*(11), 1143–1152. https://doi.org/10.1016/j.chembiol.2006.09.005

Mavi, P. S., Singh, S., & Kumar, A. (2020). Reductive Stress: New Insights in Physiology and Drug Tolerance of *Mycobacterium*. *Antioxidants & Redox Signaling*, *32*(18), 1348–1366. https://doi.org/10.1089/ars.2019.7867

Wade, Y., Daniel, R. A., & Leak, D. J. (2019). Heterologous Microcompartment Assembly in *Bacillaceae* : Establishing the Components Necessary for Scaffold Formation. *ACS Synthetic Biology*, *8*(7), 1642–1654. https://doi.org/10.1021/acssynbio.9b00155

Whitham, J. M., Tirado-Acevedo, O., Chinn, M. S., Pawlak, J. J., & Grunden, A. M. (2015). Metabolic Response of Clostridium ljungdahlii to Oxygen Exposure. *Applied and Environmental Microbiology*, *81*(24), 8379–8391. https://doi.org/10.1128/AEM.02491-15

Zhang, J.-Z., Li, Y.-Z., Xi, Z.-N., Zhang, Y., Liu, Z.-Y., Ma, X.-Q., & Li, F.-L. (2025). Inducible promoters of bacterial microcompartments improve the CRISPR/Cas9 tools for efficient metabolic engineering of *Clostridium ljungdahlii*. *Applied and Environmental Microbiology*, *91*(4). https://doi.org/10.1128/aem.02183-24
